# *De novo *assembled expressed gene catalog of a fast-growing *Eucalyptus *tree produced by Illumina mRNA-Seq

**DOI:** 10.1186/1471-2164-11-681

**Published:** 2010-12-01

**Authors:** Eshchar Mizrachi, Charles A Hefer, Martin Ranik, Fourie Joubert, Alexander A Myburg

**Affiliations:** 1 Department of Genetics, Forestry and Agricultural Biotechnology Institute (FABI), University of Pretoria, Pretoria, 0002, South Africa; 2 Bioinformatics and Computational Biology Unit, Department of Biochemistry, University of Pretoria, Pretoria, 0002, South Africa

## Abstract

**Background:**

*De novo *assembly of transcript sequences produced by short-read DNA sequencing technologies offers a rapid approach to obtain expressed gene catalogs for non-model organisms. A draft genome sequence will be produced in 2010 for a *Eucalyptus *tree species (*E. grandis*) representing the most important hardwood fibre crop in the world. Genome annotation of this valuable woody plant and genetic dissection of its superior growth and productivity will be greatly facilitated by the availability of a comprehensive collection of expressed gene sequences from multiple tissues and organs.

**Results:**

We present an extensive expressed gene catalog for a commercially grown *E. grandis *× *E. urophylla *hybrid clone constructed using only Illumina mRNA-Seq technology and *de novo *assembly. A total of 18,894 transcript-derived contigs, a large proportion of which represent full-length protein coding genes were assembled and annotated. Analysis of assembly quality, length and diversity show that this dataset represent the most comprehensive expressed gene catalog for any *Eucalyptus *tree. mRNA-Seq analysis furthermore allowed digital expression profiling of all of the assembled transcripts across diverse xylogenic and non-xylogenic tissues, which is invaluable for ascribing putative gene functions.

**Conclusions:**

*De novo *assembly of Illumina mRNA-Seq reads is an efficient approach for transcriptome sequencing and profiling in *Eucalyptus *and other non-model organisms. The transcriptome resource (Eucspresso, http://eucspresso.bi.up.ac.za/) generated by this study will be of value for genomic analysis of woody biomass production in *Eucalyptus *and for comparative genomic analysis of growth and development in woody and herbaceous plants.

## Background

Ultra-high-throughput second-generation DNA sequencing technologies from companies such as Roche (454 pyrosequencing), Illumina (sequencing by synthesis, Solexa GA) and Applied Biosystems (sequencing by ligation, SOLiD), are increasingly being used for novel exploratory genomics in small to medium-sized laboratories. "Short-read" (36 - 72 nt) technologies such as those of Illumina and Applied Biosystems have proven to be exceptionally successful in a wide variety of whole-transcriptome investigations [1-5], but most of these studies have relied on prior sequence knowledge such as an annotated genome for qualitative and quantitative transcriptome analyses.

Genome assembly of short sequences without any auxiliary knowledge has primarily utilized 454 sequencing data, due to the longer individual read lengths of 150-400 base pairs (bp). However, short-read sequencing (Illumina GA and SOLiD) has been successfully used for *de novo *assembly of small bacterial genomes (2-5 Mbp), where 36 bp reads have been assembled [6-8] and hybrid approaches, where genomes are *de novo *assembled using a combination of reads from multiple sequencing platforms to overcome the inherent limitations of each technology, have been used to successfully assemble genomes of up to 40 Mbp [9,10]. More recently, the sequencing of the giant panda genome was demonstrated [11] using *de novo *assembly of sequence derived from a single platform (Illumina), but utilizing a combination of different insert sizes, allowing assembly of an estimated 94% of the genome (2.25 Gbp). *De novo *assembly of large, highly repetitive and highly heterozygous eukaryotic genomes from short-read data remains a challenge.

In transcriptome studies, 454 pyrosequencing has proven very useful for generating ESTs representing the majority of expressed genes. This has enabled gene discovery in a variety of previously uncharacterized eukaryotic organisms with no or little *a priori *DNA sequence information [12-16]. However, relatively few published studies have attempted *de novo *assembly of whole-transcriptome sequences from short-read data such as that generated by Illumina GA or SOLiD technologies. Assembly of short (36-72 bp) read data into accurate, contiguous transcript sequences has only recently been reported [17-19] demonstrating that assembly of long, potentially full-length, transcript assemblies is indeed possible.

*Eucalyptus *tree species and hybrids presently constitute the most widely planted (≈ 20 Mha) and commercially important hardwood fibre crop in the world. They are mainly utilized for timber, pulp and paper production [20]. Their fast growth rates and wide adaptability may in future allow sustainable and cost efficient production of woody biomass for bioenergy generation [21,22]. *Eucalyptus *will soon be only the second forest plantation genus (after *Populus*) for which a reference genome sequence will be completed by end 2010 [23]. To support the genome annotation effort, there is much value in having a dataset of genes with strong transcriptional evidence across a range of tissues and developmental stages. Until recently, limited amounts of *Eucalyptus *EST/unigene data were available in public databases, mainly due to the fact that commercial interests have necessitated private EST collections [24]. As of March 2010, aside from a mixed-species collection of ≈56,000 nucleotide sequences on NCBI (≈ 37,000 of which are Sanger EST sequences) and which contain extensive redundancy, the largest effort to date to generate a comprehensive catalogue of expressed genes in a single *Eucalyptus *species was based on 454 sequencing of cDNA fragments from *E. grandis *trees [15]. While this study provided an excellent representation of expressed genes and gene ontology classes in *E. grandis*, the relatively short lengths of the assembled contigs (mean length of 389 bp for all contigs longer than 200 bp) meant that very few complete gene models were represented. There remains therefore a fundamental need for a high-quality expressed gene catalog for *Eucalyptus*, to support genome annotation efforts and discern authentically expressed genes from predicted gene models, as well as for future genomics research, which will include transcriptome, proteome and metabolome profiling.

In the process of producing such a high-quality expressed gene catalog for *Eucalyptus*, we addressed three main questions: First, is it feasible to *de novo *assemble Illumina mRNA-Seq data into contiguous, near full-length gene model sequences for *Eucalyptus*? Second, what genes make up the expressed gene catalog for a fast-growing *Eucalyptus *plantation tree? Finally, can we re-use the mRNA-Seq data to create a tissue and organ-specific digital expression profile for each assembled contig? We addressed these questions by generating a comprehensive set of expressed gene sequences from a commercially grown *Eucalyptus *hybrid (*E. grandis *× *E. urophylla*) clone using Illumina mRNA-Seq technology and *de novo *short-read assembly. We report herein the complete annotation of the expressed gene catalog based on comparative analysis with the published *Arabidopsis thaliana *[25], *Populus trichocarpa *[26] and *Vitis vinifera *[27] protein-coding datasets. We describe an interactive database of annotated transcript sequences, coding sequences (CDSs) and derived protein sequences (Eucspresso, http://eucspresso.bi.up.ac.za/, CA Hefer, E Mizrachi, AA Myburg, F Joubert, unpublished), which will be continuously updated and curated in association with the *Eucalyptus *Genome Network (EUCAGEN, http://www.eucagen.org) as part of an effort to initiate a publicly accessible database for *Eucalyptus *transcriptomics research similar to that produced for *Populus *[28].

## Results

### *De novo *assembly, validation and annotation of contigs

**Figure 1:**
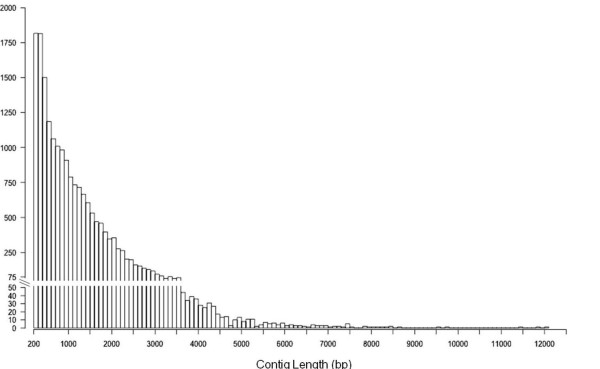
**Summary distribution of the lengths of the 18,894 assembled contigs (> 200 bp, mean length = 1170 bp, N50 = 1,640 bp, Q3 = 1,573 bp, Max = 12,053 bp)**.

In total, 62 million paired-end reads of raw mRNA-Seq data (6.90 Gbp) representing poly(A)-selected RNA from six *Eucalyptus *tissues and varying in lengths from 36 bp to 60 bp, were generated in 14 lanes on Illumina GA and GAII instruments. Following a sequence filtering process to exclude low quality and ribosomal RNA-derived reads, we assembled 36 million paired-end reads (3.93 Gbp, Additional file 1 - Table S1 and Figure S1, NCBI Sequence Read Archive accession SRA012408) of non-normalized mRNA sequence, using the Velvet short-read assembler (version 0.7.30, [29]). In total, 18,894 RNA-derived contigs were assembled (comprising 22.1 Mbp of transcriptome sequence) that were greater than 200 bp in length (mean = 1170 bp, Figure 1 and Additional file 2), with a median coverage per base (CPB) per contig of 37×, ranging from 8× (minimum coverage cut-off for assembly) to 5,262× (Additional file 1 Figure S2).

**Figure 2:**
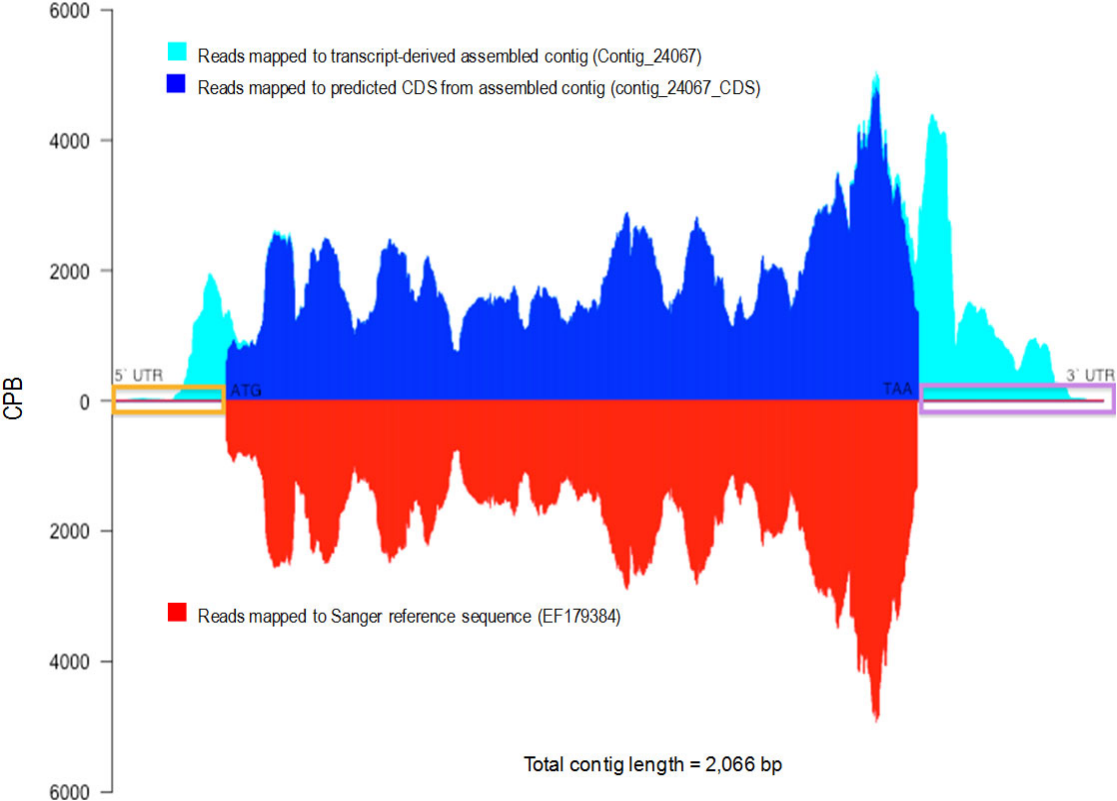
**Comparison of the *de novo *assembled contig of the *Eucalyptus grandis *UDP-glucose dehydrogenase (*UGDH*) transcript to a reference Sanger-based sequence (Genbank **EF179384**) for the same gene**. Peak height indicates coverage per base (CPB) of mapped short-reads across each sequence. CPB of the fully assembled contig is shown in cyan. CPB of the predicted CDS region is shown in dark blue. CPB of the Sanger reference sequence is shown in red. 5' UTR (orange box) and 3' UTR (purple box) regions are indicated.

We performed *ab inito *CDS prediction using GENSCAN [30] and found that 15,713 contigs (83.2%) contained a predicted CDS (Additional file 1 Table S3). Analysis of the predicted coding sequences using Anaconda [31] identified 6,208 contigs that contained putatively full-length CDSs (i.e. containing start and stop codons), 4,610 predicted to contain a start but no stop codon, 4,874 predicted to contain a stop but no start codon, and only 21 with neither. To ascertain the quality of Velvet assembly of short reads into long contiguous coding sequences, we compared a subset of 35 of our transcript-derived contigs to corresponding Sanger-sequenced, full-length, cloned *Eucalyptus grandis *mRNA sequences in NCBI (Figure 2 and Additional file 3). Paired reads were independently mapped to each Sanger reference sequence, the *de novo *assembled Velvet contig and its corresponding predicted CDS. A Needleman-Wunsch alignment of these three sequences was used for contiguity validation of the assembled contigs. Independently, each sequence had 100% coverage validation across the contig, except in cases of low quality assembly ('N's inserted by Velvet), which occurred in regions of coverage lower than 8× per base. Of the 35 transcript-derived contigs evaluated, 25 (71%) assembled completely with a 5' UTR, 3' UTR, as well as a contiguous coding sequence matching that of the reference mRNA sequence. We found several cases where, despite high coverage, our transcript-derived contigs differed from the Sanger reference sequence due to indels, but these were generally in the UTR regions and likely represent allelic differences between the F1 hybrid individual and the reference sequences (Additional file 3).

**Figure 3:**
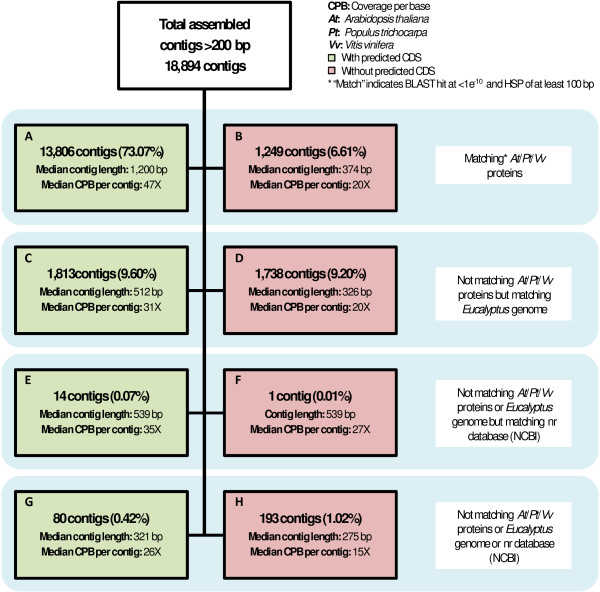
**Breakdown of annotation categories for all 18,894 transcript-derived contigs**. A large proportion (98.5%) of assembled contigs (A-D) had significant BLAST hits (< 1e^-10 ^confidence, minimum 100 bp HSP match length) to the draft *Eucalyptus *genome assembly (http://eucalyptusdb.bi.up.ac.za/), 80% of which (A, B) also exhibited significant similarity (BLASTX, < 1e^-10^, > 100 bp HSP) to coding sequences of *Arabidopsis*, *Populus *or *Vitis*.

Of the 18,894 assembled contigs, 18,606 (98.48%) exhibited significant similarity (BLASTN, < 1e^-10^, [31]) to the preliminary draft 8X DOE-JGI *E. grandis *genome assembly (http://eucalyptusdb.bi.up.ac.za/) consistent with the origin of the mRNA contigs (an F1 hybrid of *E. grandis *and *E. urophylla*). We further characterized the assembled contigs by high stringency BLASTX analysis (< 1e^-10 ^confidence, minimum 100 bp high scoring pair (HSP) match length) to protein datasets from three reference sequenced angiosperm genera (*Arabidopsis*, *Populus *and *Vitis*). Cumulatively, 15,055 contigs (79.68%) exhibited high similarity to *Arabidopsis *(14,235 contigs), *Populus *(14,769 contigs) or *Vitis *proteins (14,833 contigs, Additional file 1 Figure S3). Of the 15,055 contigs with high similarity to *Arabidopsis*, *Populus *or *Vitis *proteins, 13,806 (91.70%) also contained predicted coding sequences (Figure 3A), while 1,249 (8.30%) did not (Figure 3B), possibly due to low expression of these transcripts which would have resulted in lower coverage and shorter contigs that represented only a fraction of the open reading frame (or mostly UTR sequence). Predicted codon usage and amino acid frequencies in the proteome represented by the *Eucalyptus *expressed gene catalog were very similar to those of expressed gene catalogs from *Arabidopsis *and *Populus *(Additional file 1 Figure S4 and Figure S5).

To compare the completeness of our expressed gene catalogue to that of all publicly available gene sequence data for *Eucalyptus*, we generated a separate dataset, termed EucALL, containing all publicly available *Eucalyptus *gene sequence data to date (March 2010). This included all NCBI unigenes and ESTs, assembled 454 EST data from *E. grandis *leaf tissue (DOE-JGI, http://eucalyptusdb.bi.up.ac.za/), assembled 454 EST data produced by Novaes and colleagues [15], and the EucaWood contig dataset [33]. We compared the representation of *Arabidopsis *genes in the EucALL dataset and in our assembled *E. grandis *× *E. urophylla *(EGU) transcript dataset by BLASTX at significance levels of < 1e^-05^, < 1e^-10 ^and < 1e^-20 ^(Additional file 1 Table S2). While the overall numbers of hits were higher in the EucALL dataset, these were mostly in the lower size ranges. For our *de novo *assembled contigs, a much higher number of significant hits in contigs larger than 2000 bp in size (6,602 compared to 1,940 at significance < 1e^-10^) suggested that a greater proportion of our contigs represent full-length gene models than the publicly available *Eucalyptus *gene sequence set (EucALL).

### Functional annotation of the expressed gene catalog

**Figure 4:**
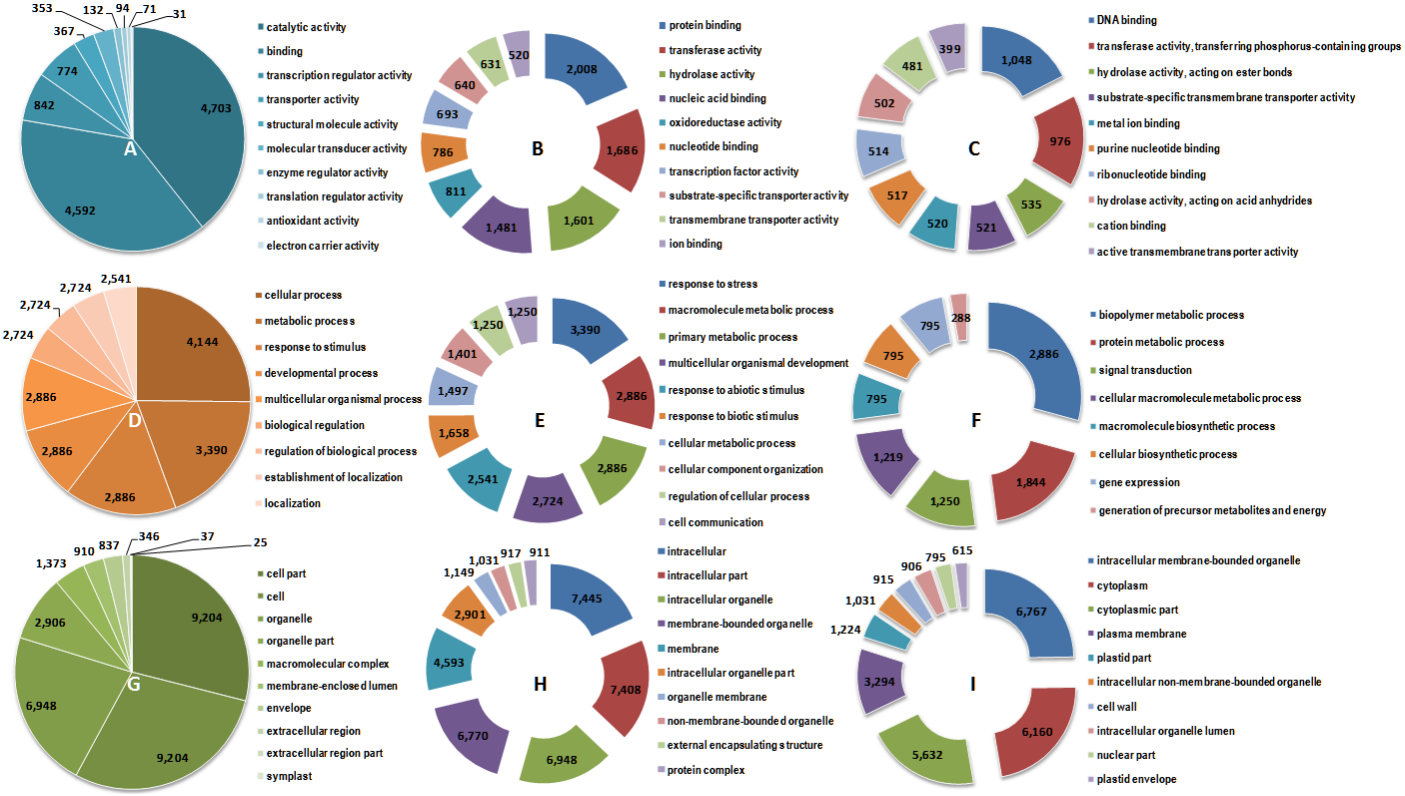
**Top ten most represented GO categories under the "Molecular Function" (A-C), "Biological Process" (D-F) and "Cellular Compartment" (G-I) categories in level 2 (A, D and G), 3 (B, E and H) and 4 (C, F and I)**. The numbers and proportions in all categories reflect the diversity and complexity of genes expressed in multiple tissues sampled to make up the *Eucalyptus *gene catalog.

The transcript-derived contig sequences were annotated according to several functional annotation conventions, including Gene Ontology (GO - http://www.geneontology.org/), KEGG (http://www.genome.jp/kegg/) and InterProScan (http://www.ebi.ac.uk/Tools/InterProScan/). The numbers and assortment of allocated GO categories provides a good indication of the large diversity of expressed genes sampled from the *Eucalyptus *transcriptome (Figure 4). This was also reflected in the diversity of InterProScan categories identified (Additional file 1 Figure S6 and Figure S7), as well as the comprehensive coverage of biochemical processes by KEGG annotation, which was similar to that of the entire *Arabidopsis *gene catalog (Additional file 1 Figure S8).

### Digital expression profiling

An accepted method of identifying large scale differences in gene expression is to use EST abundance as an indicator of transcript abundance. This method has been implemented and validated in numerous studies using Sanger-derived ESTs [34,35], as well as 454-pyrosequencing methods [13,36-39]. Quantitative transcriptome analysis using ultra-high-throughput sequencing technologies such as Illumina and SOLiD has been shown to be accurate and highly correlated with other quantitative methods such as RT-qPCR and microarray analysis [1,5]. To quantify tissue-specific transcript abundance reflected in our short-read dataset, we combined data (multiple lanes in most cases) generated from the same tissues and mapped six tissue-specific datasets (Additional file 1 Table S1) to the assembled gene catalog using Bowtie [40]. Following this, we used the Cufflinks [41] program (http://cufflinks.cbcb.umd.edu), which provides relative abundance values by calculating Fragments Per Kilobase of exon per Million fragments mapped (FPKM) as validated previously [2]. This enabled the allocation of a tentative digital expression profile for each transcript-derived contig (Additional file 4).

**Figure 5:**
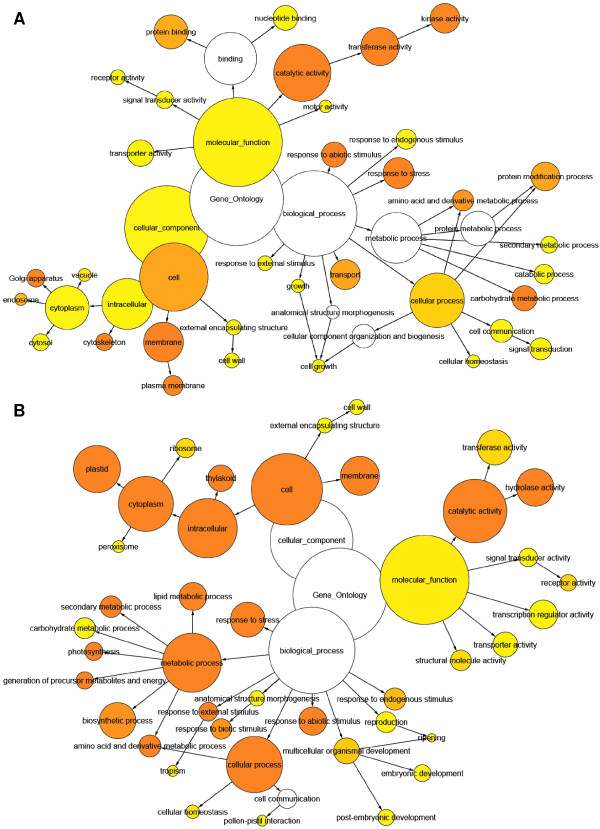
**Over-represented GO categories in xylem (A - 1,897 annotated contigs) and leaf (B - 1,531 annotated contigs) tissues**. All genes with a FPKM value more than two-fold higher in one tissue type versus the other were considered for the analysis. Data were analyzed using BiNGO (Maere et al. 2005). Node size is proportional to the number of genes in each category and colors shaded according to significance level (white - no significant difference, yellow - FDR = 0.05, Orange - FDR < 0.05).

To compare between two general tissue types that are of interest for woody biomass production, we evaluated groups of genes whose FPKM values were greater than two-fold higher in woody (xylogenic) tissues (average FPKM of immature xylem and xylem: 1,897 annotated contigs) or leaf (non-xylogenic) tissues (average FPKM of shoot tips, young leaves and mature leaves: 1,531 annotated contigs). GO categories over-represented in the xylem-upregulated set compared to the leaf set (Figure 5A) was representative of developing woody tissues, with significant enrichment (*p *< 0.05) in signalling ("kinase activity"), carbohydrate metabolism, and genes associated with the Golgi, cytoskeleton and the plasma membrane - consistent with an emphasis on delivery of biopolymers to the cell wall. In contrast, gene categories significantly enriched (*p *< 0.05) in leaf tissue compared to woody tissue (Figure 5B) were associated with photosynthesis ("plastid", "thylakoid", "photosynthesis"), growth and energy production (precursor metabolites, "lipid biosynthesis", "amino acid metabolism").

We also interrogated our transcriptome data using the "core xylem gene set" identified in *Arabidopsis *by Ko and colleagues [42]. Of the 52 genes identified by the authors as markers of secondary xylem formation in *Arabidopsis*, 33 had putative homologues in the *Eucalyptus *transcriptome (BLASTX, < 1e^-10^) and in total 43 contigs were identified. Of these, 40 (93%) showed greater than two-fold "Xylem" to "Leaf" digital expression profile ratios and six were only detected in xylem tissues (Additional file 1 Table S4). Most of the expression profiles were also highly correlated with that of secondary cell wall-specific *Eucalyptus *cellulose synthase genes, similar to the patterns previously observed in *Arabidopsis*. These results are comparable to the 80% (51 out of 63 genes) reported recently for the same set of *Arabidopsis *homologs in *Populus *[43], which provided further support for the biological validity of the short-read-based digital expression profiles associated with the *Eucalyptus *expressed gene catalog.

### Public data resource

We constructed a public data resource, Eucspresso (http://eucspresso.bi.up.ac.za), which provides a searchable interface to the assembled contigs. The database can be queried based on closest homologous entry in the *Arabidopsis thaliana *(TAIR9), *Populus trichocarpa *(Version 2.0) and *Vitis vinifera *(Sept 2009 build) sequence data sets. Simple and compound keyword searches can be performed based on all of the functional annotation terms and the predicted coding and protein sequences can be obtained for all contigs. Finally, the tissue-specific (FPKM) digital expression profile and the location of each contig in the draft 8X *E. grandis *genome assembly (http://eucalyptusdb.bi.up.ac.za/) can be viewed from within Eucspresso.

## Discussion

We have assembled nearly 19,000 expressed gene sequences from xylogenic and non-xylogenic tissues of an actively growing *Eucalyptus *plantation tree using only Illumina mRNA-Seq technology and *de novo *short-read assembly. Quality control comparisons to full-length, cloned, Sanger-derived transcript sequences from *Eucalyptus*, as well as multiple lines of evidence such as CDS prediction and Pfam prediction showed that the transcript assemblies are robust and that thousands of full-length coding sequences and their respective 5' and/or 3' UTR regions were successfully assembled. Comparison of assembled gene models to gene catalogs of other angiosperm species by BLAST analysis and functional annotation (GO, InterProScan and KEGG category numbers and proportions, Figure 4 and Additional file 1 - Figure S6, Figure S7 and Figure S8) indicate that we have sampled an expansive and diverse expressed gene catalog representing a large proportion of the genes expressed in mature *Eucalyptus *trees across a variety of woody and non-woody tissues. Comparison to all publicly available *Eucalyptus *DNA sequence suggests that we have sampled a more comprehensive set of genes, which is also more complete in length (Additional file 1 - Table S2) from a single eucalypt tree genotype than has been available to date for the entire genus. Additionally, using a validated approach to quantify mRNA-Seq data we have produced an informative database of transcript abundance across six *Eucalyptus *tree tissues, which, due to the depth of sequencing, results in higher sensitivity and wider dynamic range than Sanger or 454-derived EST counts usually associated with this type of analysis.

A concern associated with *de novo *assembly of transcript sequences, be it Sanger derived [33] or 454 sequence derived [15] assemblies, is the contiguity of assembled sequences. This concern intuitively increases as the read length decreases, and may be one of the main reasons why most transcriptome *de novo *assembly approaches have utilized technologies with longer read lengths to date. We provide several lines of evidence which jointly support the contiguity of transcript sequences assembled in our study using short-read data. First, a high proportion of the contigs exhibited high-confidence BLASTX similarity to protein sequences from annotated gene catalogs of three angiosperm species *Arabidopsis*, *Populus *and *Vitis *(Figure 3). Second, a large proportion of the contigs contained long, near full-length, predicted CDSs (Figure 3). Third, InterproScan analysis predicted 45,687 protein domains, which is indicative of contiguous, in-frame predicted protein sequences (Additional File 1). Finally, a random subset of the contigs, which represented a variety of length and read coverage, were validated by direct alignment to previously published, Sanger sequenced, full-length *Eucalyptus *genes that were directly cloned from cDNA (Additional File 3).

Assigning biological significance to *de novo *assembled contigs should be approached with caution. In our study, 13,806 assembled gene models (73.07% of the total assembled contigs, Figure 3A) were considered high confidence annotations due to the presence of a significant high stringency BLAST hit in other angiosperm species, as well as a predicted CDS. These contigs had relatively high coverage per base (CPB) values (median 47X) as compared to contigs lacking a predicted CDS (median CPB of 20× or lower, Figure 3B and 3D and Supplemental Table S3). Thus, a lack of CDS prediction was generally associated with low gene expression level and low CPB, which resulted in 'N's inserted by Velvet in the contig sequences (Figure 3B and 3D and Supplemental Table S3). The assembly quality and annotation of these sequences could be improved in future by even deeper sequencing and the addition of data from new tissue types. Another possible source of error is the spurious prediction of CDSs in long, non-coding RNAs, which has been previously shown to occur [44,45]. It is notable that of the 1,813 *Eucalyptus*-derived contigs with no significant BLAST hit to other angiosperms, but containing a predicted CDS (Figure 3C), only 81 contigs had predicted InterProScan domains. Additionally, the median CDS to contig length ratio was 0.33, as compared to 0.62 in the 13,806 high confidence contigs in Figure 3A, which suggests that many of these CDS predictions may be false positives. *De novo *assembled transcriptome datasets lack the ability to distinguish and classify the lower confidence annotations, an exercise that is beyond the scope of this study, albeit one that can be resolved once a genome-based predicted set of gene models is available.

Validation of the digital expression (FPKM) profiles using the "core xylem gene set" identified in *Arabidopsis *[42] has precedence in similar investigations in conifers [46], cotton [47] and poplar [43]. This analysis, combined with the results shown in Figure 5A and Figure 5B, lend support to the biological significance of digital expression profiles derived from short-read sequencing technology, which will assist in the discovery and annotation of novel *Eucalyptus *genes - and using the genome sequence, promoters - playing key roles in growth and development, and particularly in woody biomass production. The Eucspresso online resource produced from this study, as well as future comparative analysis with other woody species such as *Vitis *and *Populus*, will be valuable for studying the unique biology of woody perennials.

## Conclusions

Taking into consideration the number, length, coverage and quality of assembled gene models, as well as their digital expression profiles, this dataset surpasses several previous *de novo *transcriptome assemblies using Illumina [17,18] or 454 technology [13-16]. This can primarily be attributed to the amount of data generated (3.93 Gbp of non-rRNA derived reads), the diversity of tissues sampled and strategy of paired-end sequencing, as well as read-length (mostly 50-60 bp, compared to only 36 bp in earlier studies). Our dataset was generated using several generations of Illumina GA technology, but considering the current throughput of Illumina sequencing (up to 100 Gbp per flowcell), a gene catalog of this scale can now be produced using a single lane of Illumina mRNA-Seq. Finally, non-normalized short-read data will be extremely useful for downstream applications such as digital gene expression profiling and detection of alternative transcript structure, once reference models are available from the genome.

## Methods

### Plant tissue collection

Tissues from a six-year-old ramet of a commercially grown *E. grandis *× *E. urophylla *hybrid clone (GUSAP1, Sappi Forestry, Kwambonambi, South Africa) were collected in a clonal field trial and immediately frozen in liquid nitrogen, as previously described by Ranik and Myburg [48]. The following tissues were sampled from approximately breast height (1.35 m) on the main stem following bark removal: immature xylem (outer glutinous 1-2 mm layer comprising early developing xylem tissue) and xylem (after removal of the immature xylem layer, 2-mm-deep planing including xylem cells in advanced stages of maturity). Early developing phloem tissue including small amounts of cambial cells was collected by scraping the first 1-2 mm layer from the inner surface of the bark. Additionally, we sampled shoot tips (soft green termini of young crown tip branches containing shoot primordia and apical meristems), young leaves (rapidly-growing leaves in the process of unfolding) and mature leaves (older, fully expanded leaves of the current growth season).

### Paired-end mRNA-Seq library preparation and sequence generation

Total RNA was extracted from the six tissues using the protocol described previously [49]. Total RNA quality and concentration were determined using the Agilent RNA 6000 Pico kit (Agilent, Santa Clara, CA) on a 2100 Bioanalyzer (Agilent). Enrichment of polyA+ RNA was performed using the Oligotex midi kit (Qiagen, Valencia, CA). Two hundred nanograms of polyA+ RNA were fragmented in 1× RNA fragmentation solution (Ambion, Austin, TX) at 70°C for 5 minutes. The fragmented RNA was precipitated with three volumes of ethanol and re-dissolved in water. Double-stranded cDNA was synthesized using the cDNA Synthesis System (Roche, Indianapolis, IN) according to manufacturer's instructions using random hexamers (Invitrogen, Carlsbad, CA) to prime the first strand cDNA synthesis. Paired-end libraries with approximate average insert lengths of 200 base pairs were synthesized using the Genomic Sample Prep kit (Illumina, San Diego, CA) according to manufacturer's instructions. Prior to cluster generation, library concentration and size were assayed using the Agilent DNA1000 kit (Agilent) on a 2100 Bioanalyzer (Agilent). Libraries were sequenced on a Genome Analyzer equipped with a paired-end module (versions I, II and IIx, Illumina).

### *De novo *assembly of mRNA-Seq data

After removing sequences containing low quality bases ('N's) or single base repeats and ribosomal RNA sequences, the 3.93 Gbp dataset was used for assembly and subsequent coverage per base (CPB) estimation for each assembled contig. We assembled the filtered Illumina paired-end (PE) reads using Velvet version 0.7.30 [29]. Previous studies [1-3,50] have demonstrated that mRNA-Seq technology produces uneven coverage over a transcript, which prompted us to follow a coverage-assisted reference assembly strategy. Using Mosaik (http://bioinformatics.bc.edu/marthlab/Mosaik) to align the filtered Illumina PE sequences to the assembled contigs, the average coverage per contig was calculated. A custom script was then developed to extract the pairs of sequences that mapped to each contig, and using that contig as a template, each contig was re-assembled using Velvet with the associated expected coverage parameter set to the Mosaik average coverage value for that contig.

### Contig validation

The degree to which the assembled contigs represented long, contiguous RNA transcript sequences, was evaluated by aligning 35 Velvet contigs and their respective predicted CDSs to full-length, cloned, Sanger-derived *Eucalyptus *reference sequences present in NCBI. CPB was calculated for the sequences using BWA [51] and a global pairwise alignment of the sequences was performed using the Needle package from EMBOSS [52]. Plots were constructed from the alignments with the CPB on the y-axis of the plot. Zero coverage values were assigned to gaps in the alignments. This revealed where gaps and/or potentially misassembled regions were present in the assembled contigs, and to what depth these contigs were sequenced.

### Coding sequence prediction

Coding sequence predictions were performed using GENSCAN [30] and AUGUSTUS [53], predicting 15,713 and 15,904 proteins respectively. The difference in coding sequences predicted could be attributed to the different training data sets used and inherent difficulty of predicting coding sequences from incomplete genomic sequences. The GENSCAN results (15,713 predicted proteins) were used in downstream analyses.

### Annotation of assembled contigs

Homology searches were performed against public sequence databases. The newest versions as of February 2010 of the protein sequences of *Arabidopsis *(TAIR 9), *Vitis *(Sept 2009 build) and *Populus *(version 2.0, Phytozome) were used to construct the individual BLAST datasets. The *Eucalyptus *public dataset (EucAll) consisted of 45,442 entries in Genbank (downloaded March 2010), 13,930 entries from the *Eucalyptus *Wood unigenes and ESTs [33], *E. grandis *leaf tissue ESTs (120,661 entries from DOE-JGI-produced 454 sequences, http://eucalyptusdb.bi.up.ac.za/) and 190,106 Unigenes and singlets from *E. grandis *454 data [15]. The BLAST e-value threshold was set at 1e^-10^, with a minimum alignment length of 100 nucleotides (33 amino acids). Functional annotation (GO and KEGG) was performed using BLAST2GO [54], using the default annotation parameters (BLAST e-value threshold of 1e^-06^, Gene Ontology annotation threshold of 55). InterPro annotations were performed using InterProScan (http://www.ebi.ac.uk/Tools/InterProScan/).

### Coverage and FPKM determination

Sequence depth and base coverage were calculated using BWA (Lin et al. 2009) and the FPKM values estimated by aligning the Illumina reads to the assembled transcriptome using Bowtie [40] and estimating the expression level of each predicted transcript (FPKM value) using Cufflinks (http://cufflinks.cbcb.umd.edu) [41].

## Authors' contributions

EM drafted the manuscript, helped sample the material, prepared the libraries, participated in the *de novo *assembly and data analysis, and helped design Eucspresso. CAH performed the *de novo *assembly and automated annotation, participated in data analysis, designed the database Eucspresso, and helped draft the manuscript. MR prepared the libraries, helped sample the material and participated in data analysis. FJ participated in data analysis and the design of Eucspresso. AAM conceived of the study, and participated in its design and coordination and helped to draft the manuscript and participated in data analysis, and helped design Eucspresso. It is the authors' opinion than EM and CAH contributed equally as first authors to this manuscript. All authors have read and approved the final version of the manuscript.

## Supplementary Material

Additional file 1 Supplemental Tables S1-S3 and Supplemental Figures S1-S8 referred to in text. Click here for file

Additional file 2 **FASTA formatted sequences of all 18,894 assembled contigs**. Click here for file

Additional file 3 **Contig validation, Needleman-Wunsch alignment figures**. Click here for file

Additional file 4 **Table containing all 18,894 contig names and calculated FPKM values for six tissues (immature xylem, xylem, phloem, shoot-tips, young leaves and mature leaves)**. Eucspresso (http://eucspresso.bi.up.ac.za/) - Online database with mRNA contig sequences and their Blast, GO, KEGG, Pfam annotations. The short-read sequence data have been submitted to the NCBI Sequence Read Archive (http://www.ncbi.nlm.nih.gov/sra) under accession SRA012408. Click here for file
