# Supplementary material for: De novo assembled expressed gene catalog of a fast-growing Eucalyptus tree produced by Illumina mRNA-Seq
Source: BMC Genomics. 2010 Dec 1;11:681. doi: 10.1186/1471-2164-11-681 (PMC3053591; doi:10.1186/1471-2164-11-681)
Supplement: Additional file 1 — Supplemental Tables S1-S3 and Supplemental Figures S1-S8 referred to in text. [file 1471-2164-11-681-S1.DOC]

# *De novo* assembled expressed gene catalog of a fast-growing *Eucalyptus* tree produced by Illumina mRNA-Seq

Eshchar Mizrachi^1*^, Charles A Hefer^2*^, Martin Ranik^1^, Fourie Joubert^2^ and Alexander A Myburg^1§^

*These authors contributed equally to this work

^1^ Department of Genetics, Forestry and Agricultural Biotechnology Institute (FABI), University of Pretoria, Pretoria, 0002, South Africa

^2^ Bioinformatics and Computational Biology Unit, Department of Biochemistry, University of Pretoria, Pretoria, 0002, South Africa

^§^Corresponding author

## Additional file 1 - Supplemental Tables and Figures

**Supplemental Table S1.** Summary of filtered RNA-Seq data generated for *de novo* transcriptome assembly. Fourteen RNA-Seq libraries were prepared and sequenced from RNA derived from six tissues of a *Eucalyptus grandis* x *E. urophylla* F1 hybrid clone and filtered to exclude low quality and ribosomal RNA-derived reads.

**Supplemental Table S2.** The complete gene catalog from *Arabidopsis* *thaliana* (TAIR 9) was used to query the EucALL dataset and the Velvet assembled dataset containing 18,894 contigs from this study. Significant BLAST hits were counted in incrementing contig size classes from the target datasets. In the Velvet-assembled dataset, the number of significant hits was 3.4 times higher in size categories greater than 2000 bp in length, indicating that the Velvet assembled contigs contained more full-length gene models than current publicly available coding sequence for *Eucalyptus*.

**Supplemental Table S3.** Summary of *de novo* assembly statistics for different classes of annotated contigs. *At – Arabidopsis thaliana*, *Pt – Populus trichocarpa*, *Vv – Vitis Vinifera.* A positive “match” indicates a positive BLAST hit at <1e-10 and HSP of at least 100 bp in length.

**Supplemental Table S4**. List of 43 transcript-derived contigs homologous to 33 of the 52 “Core xylem genes” identified by Ko et al.[1] in *Arabidopsis* and their relative xylem to leaf FPKM ratio. In most cases, the expression profiles of these genes were highly positively correlated with that of *EgCesA1* (Contig268), a secondary cell wall-specific cellulose synthase gene [2].

**Supplemental Figure S1.** Summary of whole-transcriptome analysis strategy. RNA was isolated from six tissues (Supplemental Table S1) of a *Eucalyptus grandis x E. urophylla* F1 hybrid clone. Tissue-specific Illumina RNA-Seq libraries were paired-end (PE) sequenced to generate a total of 6.9 Gbp of raw sequence. After filtering out ribosomal RNA derived and low quality reads, ≈36 million paired-end reads (3.93 Gbp) were *de novo* assembled using Velvet (version 0.7.30,[3]) and a round of contig extension using custom scripts as explained in the METHODS section. Assembly quality was investigated by mapping reads to- and aligning a subsection of assembled contigs with their corresponding full-length reference Sanger gene sequences from NCBI, to evaluate assembly contiguity. Annotation was carried out by high stringency BLAST query of gene catalogs from three sequenced angiosperm species – *Arabidopsis thaliana*, *Populus trichocarpa* and *Vitis vinifera*. Functional annotation was performed by assigning Gene Ontology (GO - http://www.geneontology.org/), KEGG (http://www.genome.jp/kegg/) and InterProScan (http://www.ebi.ac.uk/interpro/) terms to each contig. A tissue-specific FPKM value for each contig was calculated using Cufflinks [4]. All data was integrated into an interactive database, Eucspresso (http://eucspresso.bi.up.ac.za).

**Supplemental Figure S2.** Average coverage of transcript-derived short-read contigs. Coverage per base (CPB) was calculated across each contig and a frequency histogram constructed from the coverage values of all contigs (Median CPB = 37X, Q3 = 72X, Max CPB = 5,262X).

**Supplemental Figure S3.** High stringency BLAST analysis (<1e-10 confidence blastx, minimum 100 bp HSP match length) of the *Eucalyptus* transcript-derived contigs against protein datasets from three reference sequenced angiosperm genera (*Arabidopsis*, *Populus* and *Vitis*). In total, 15,505 contigs (82.06% of the total contig dataset) exhibited similarity to *Arabidopsis* (14,231 contigs), *Populus* (14,769 contigs) or *Vitis*  (14,833 contigs), while 3,552 did not show similarity to any of the three protein datasets at the chosen confidence threshold. A core set of 14,061 (74.4%) exhibited high similarity to all three protein sets.

**Supplemental Figure S4.** Codon usage histogram for predicted coding sequences in the *Eucalyptus grandis* x *E. urophylla* hybrid (A), *Arabidopsis thaliana* (B) and *Populus tricocarpa* (C) gene catalogs. Rare codons (<5%) are highlighted in blue. Analysis was performed using Anaconda 1.5 [5]. The y-axis shows frequency of codon usage.

**Supplemental Figure S5.** Amino acid frequencies in the predicted proteomes of *Arabidopsis thaliana* and *Populus trichocarpa*, as compared to the predicted proteins from the expressed gene catalog of the *Eucalyptus grandis* x *E. urophylla* F1 hybrid. Analysis was performed using Anaconda 1.5 [5].

Supplementary Figure S6. Comparison of the 25 most abundant InterProScan categories present in the *Eucalyptus* gene catalogue (left) and their relative abundance in the complete *Arabidopsis* predicted protein coding gene catalog (right). We annotated 45,687 domains by InterProScan in the *de novo* assembled *Eucalyptus* transcribed dataset (18,894 assembled contigs, 22.1 Mbp), as compared to 158,159 domains annotated in the complete TAIR 9 predicted coding gene dataset (39,640 genes, 87 Mbp).

**Supplementary Figure S7.** Summary InterProScan statistics of the top 25 most populated categories in all domain based annotation of 18,894 *de novo* assembled contigs from *Eucalyptus*. Numbers in the centre indicate the total number of domains identified for each annotation type. Details and links to InterProScan scanning methods and member databases can be obtained at ftp://ftp.ebi.ac.uk/pub/software/unix/iprscan/README.html#7).

**Supplemental Figure S8.** Biochemical pathways represented in the *de novo* assembled gene catalog. *Arabidopsis* accessions were obtained for all *Eucalyptus* genes with a significant BLAST hit (<1e-10, minimum HSP of 100 bp), and plotted onto a biochemical pathways map using the KEGG resource (http://www.genome.jp/kegg/tool/color_pathway.html). Red edges indicate coverage of one or more genes in pathways represented in the *Eucalyptus* gene catalog that are shared with *Arabidopsis*, while green edges highlight the remaining *Arabidopsis* pathways not represented in the *Eucalyptus* gene catalog.

### Supplemental Table S1.

| **Tissue Type*** | **Dataset**^†^ | **Reads** | **Read Length**^‡^ (bp) | **Total bp (Raw Data)** |
| --- | --- | --- | --- | --- |
| Xylem | ZMSR1 | 2,568,500 | 36-38 | 95,034,500 |
| Xylem | ZMSR2 | 6,288,462 | 50-55 | 330,144,255 |
| Immature Xylem | ZMSR3 | 2,228,286 | 36-38 | 82,446,582 |
| Immature Xylem | ZMSR4 | 2,961,422 | 36-38 | 109,572,614 |
| Immature Xylem | ZMSR5 | 3,243,376 | 50-55 | 170,277,240 |
| Immature Xylem | ZMSR6 | 6,567,176 | 60-60 | 394,030,560 |
| Immature Xylem | ZMSR7 | 6,799,600 | 60-60 | 407,976,000 |
| Phloem | ZMSR8 | 6,875,592 | 50-55 | 360,968,580 |
| Shoot Tips | ZMSR9 | 3,291.364 | 50-55 | 172,796,610 |
| Shoot Tips | ZMSR10 | 8,263,698 | 60-60 | 495,821,88 |
| Shoot Tips | ZMSR11 | 8,223,074 | 60-60 | 493,384,440 |
| Young Leaf | ZMSR12 | 7,324,568 | 60-60 | 439,475,160 |
| Young Leaf | ZMSR13 | 3,650,916 | 50-55 | 191,673,090 |
| Mature Leaf | ZMSR14 | 3,466,122 | 50-55 | 181,971,405 |
| **TOTAL** |  | **71,752,174** |  | **3,925,572,916** |

*See METHODS section in main paper for sampling details. For FPKM calculations (Supplemental file SF3), six tissue-specific datasets were created by combining reads that were derived from the same tissue type.

^†^All raw data is available on NCBI SRA under accession SRA012408.

^‡^All sequencing was paired-end, with pairs ranging from 300-320 bp apart.

### Supplemental Table S2.

|  |  | **EucAll** | | | | | | **Velvet-Assembled Contigs** | | | | | |
| --- | --- | --- | --- | --- | --- | --- | --- | --- | --- | --- | --- | --- | --- |
|  |  | **>200bp** | **>300bp** | **>500bp** | **>1,000bp** | **>2,000bp** | **>3,000bp** | **>200bp** | **>300bp** | **>500bp** | **>1,000bp** | **>2,000bp** | **>3,000bp** |
| ***Arabidopsis*** | **1e-05** | 27,939 | 27,396 | 25,593 | 17,245 | 2,002 | 199 | 26,854 | 26,020 | 24,512 | 18,516 | 6,862 | 2,177 |
|  | **1e-10** | 26,587 | 26,202 | 24,662 | 16,903 | 1,940 | 199 | 25,538 | 24,757 | 23,390 | 17,744 | 6,602 | 2,114 |
|  | **1e-20** | 24,302 | 24,129 | 23,093 | 16,279 | 1,865 | 191 | 23,242 | 22,545 | 21,485 | 16,569 | 6,185 | 1,978 |

### Supplemental Table S3.

|  | **Subset of Reads** | **Number of Contigs** | **Median Contig Length (BP)** | **Median Coverage Per Base (CPB)** | **% Contigs Containing 'N's**^†^ | **Median Length of Contigs Containing 'N's (bp)** | **Median % Ns in Contigs Containing 'N's** | **Median CPB in contigs containing 'N's** |
| --- | --- | --- | --- | --- | --- | --- | --- | --- |
| **TOTAL DATASET OF ASSEMBLED CONTIGS** | **With CDS*** | 15,713 | 1,090 | 44X | 42.26% | 1,369 | 1.89% | 44X |
|  | **Without CDS** | 3,181 | 333 | 20X | 41.62% | 364 | 15.12% | 19X |
| **Matching At/Pt/Vv proteins** | **With CDS** | 13,806 | 1,200 | 47X | 43.20% | 1,453 | 1.79% | 19X |
|  | **Without CDS** | 1,249 | 374 | 20X | 40.72% | 429 | 12.17% | 20X |
| **Not matching Angiosperm proteins but Matching *Eucalyptus* genome**^‡^ | **With CDS** | 1,813 | 512 | 31X | 35.21% | 684 | 3.88% | 33X |
|  | **Without CDS** | 1,738 | 326 | 20X | 39.01% | 357 | 15.03% | 19X |
| **Not Matching *Eucalyptus* genome but Matching NR** | **With CDS** | 14 | 539 | 35X | 42.86% | 680 | 3.49% | 37X |
|  | **Without CDS** | 1 | 535 | 27X | 0.00% | NA | NA | NA |
| **Not matching *Eucalyptus* genome or NR** | **With CDS** | 80 | 321 | 26X | 40.00% | 336 | 16.94% | 25X |
|  | **Without CDS** | 193 | 275 | 15X | 41.45% | 278 | 25.47% | 17X |

*CDS predicted by GenScan analysis [6]

^†^Any contig containing at least 1 ‘N’ in its sequence was counted.

^‡^Draft 8X assembly, (http://eucalyptusdb.bi.up.ac.za/)

### Supplemental Table S4.

| **At accession** | **Description** | **Contig*** | ***EgCesA1* Expression correlation**^†^ | **Xylem/Leaf FPKM ratio**^‡^ |
| --- | --- | --- | --- | --- |
| AT1G09610 | Unknown protein | contig368 | 1.000 | 362.027 |
| AT3G15050 | IQD10 | contig10671 | 0.999 | No Leaf Expression Detected |
| AT5G03170 | FLA11 | contig2707 | 0.981 | 122.993 |
| AT1G27440 | IRX10 | contig3811 | 0.999 | 126.595 |
| AT4G18780 | AtCesA8 | contig268 | 1.000 | 57.736 |
| AT5G17420 | AtCesA7 | contig31 | 1.000 | 62.810 |
| AT1G22480 | Plastocyanin-like domain-containing protein | contig6482 | 0.891 | 68.784 |
| AT5G67210 | unknown protein | contig3195 | 0.967 | 51.224 |
| AT3G62020 | GLP10 | contig25018 | 0.937 | 36.847 |
| AT2G03200 | Aspartyl protease family protein | contig453 | 0.988 | 42.946 |
| AT2G37090 | IRX9 | contig5622 | 0.994 | 47.563 |
| AT5G44030 | AtCesA4 | contig2805 | 1.000 | 31.625 |
| AT5G54690 | IRX8 | contig1569 | 0.98 | 34.588 |
| AT4G28380 | Leucine-rich repeat family protein | contig29940 | 0.794 | 221.689 |
| AT5G01360 | Unknown protein | contig8107 | 0.931 | No Leaf Expression Detected |
| AT5G15630 | IRX6 | contig1665 | 0.99 | 32.632 |
| AT5G67210 | Unknown protein | contig5930 | 0.993 | 23.401 |
| AT4G17220 | MAP70-5 | contig7003 | 0.666 | No Leaf Expression Detected |
| AT4G22680 | MYB85 | contig3124 | 0.749 | 44.273 |
| AT5G40020 | Pathogenesis-related thaumatin family protein | contig22035 | 0.851 | 18.973 |
| AT1G27920 | MAP65-8 | contig3070 | 0.891 | 16.325 |
| AT1G63910 | MYB103 | contig16135 | 0.886 | No Leaf Expression Detected |
| AT4G27435 | Unknown protein | contig949 | 0.981 | 10.151 |
| AT2G46770 | NST1 | contig44541 | 0.918 | 78.270 |
| AT5G60720 | Unknown protein | contig7972 | 0.935 | 17.281 |
| AT3G18660 | PGSIP1 | contig19436 | 0.959 | 20.325 |
| AT5G03170 | FLA11 | contig3257 | 0.79 | No Leaf Expression Detected |
| AT5G01360 | Unknown protein | contig5954 | 0.928 | 14.072 |
| AT1G79620 | Leucine-rich repeat transmembrane protein kinase, putative | contig53943 | 0.932 | 8.553 |
| AT1G31720 | Unknown protein | contig24491 | 0.922 | 11.887 |
| AT4G28500 | SND2 | contig2382 | 0.935 | 11.174 |
| AT4G33330 | PGSIP3 | contig14715 | 0.94 | 9.997 |
| AT1G24030 | Protein kinase family protein | contig20741 | 0.704 | 29.393 |
| AT1G19300 | PARVUS | contig26681 | 0.964 | 6.764 |
| AT1G09440 | Protein kinase family protein | contig6469 | 0.969 | 10.219 |
| AT5G61340 | Unknown protein | contig41480 | 0.623 | No Leaf Expression Detected |
| AT1G66230 | MYB20 | contig10425 | 0.822 | 5.910 |
| AT4G28500 | SND2 | contig21083 | 0.577 | 13.925 |
| AT2G46770 | NST1 | contig21412 | 0.276 | 33.989 |
| AT1G33800 | Unknown protein | contig4765 | 0.734 | 2.778 |
| AT1G80170 | polygalacturonase, putative / pectinase, putative | contig2977 | -0.469 | 1.002 |
| AT5G67210 | Unknown protein | contig53083 | -0.85 | 0.130 |
| AT5G05390 | LAC12 | contig92451 | -0.624 | No Xylem Expression Detected |

* Contig (node) numbers originally assigned by Velvet during the short-read assembly. The complete list of transcript-derived contigs is available in Supplementary file SF3.

^†^ Correlation of digital expression profile to that of *EgCesA1* (Contig268 - orthologous to *AtCesA8*), a secondary cell wall associated cellulose synthase gene.

^‡^ Ratio of the average FPKM value for xylem and immature xylem to the average for shoot tips, young leaves and mature leaves. Cases where no xylem or no leaf expression were detected (average FPKM = 0) are indicated.

### Supplemental Figure S1.

**
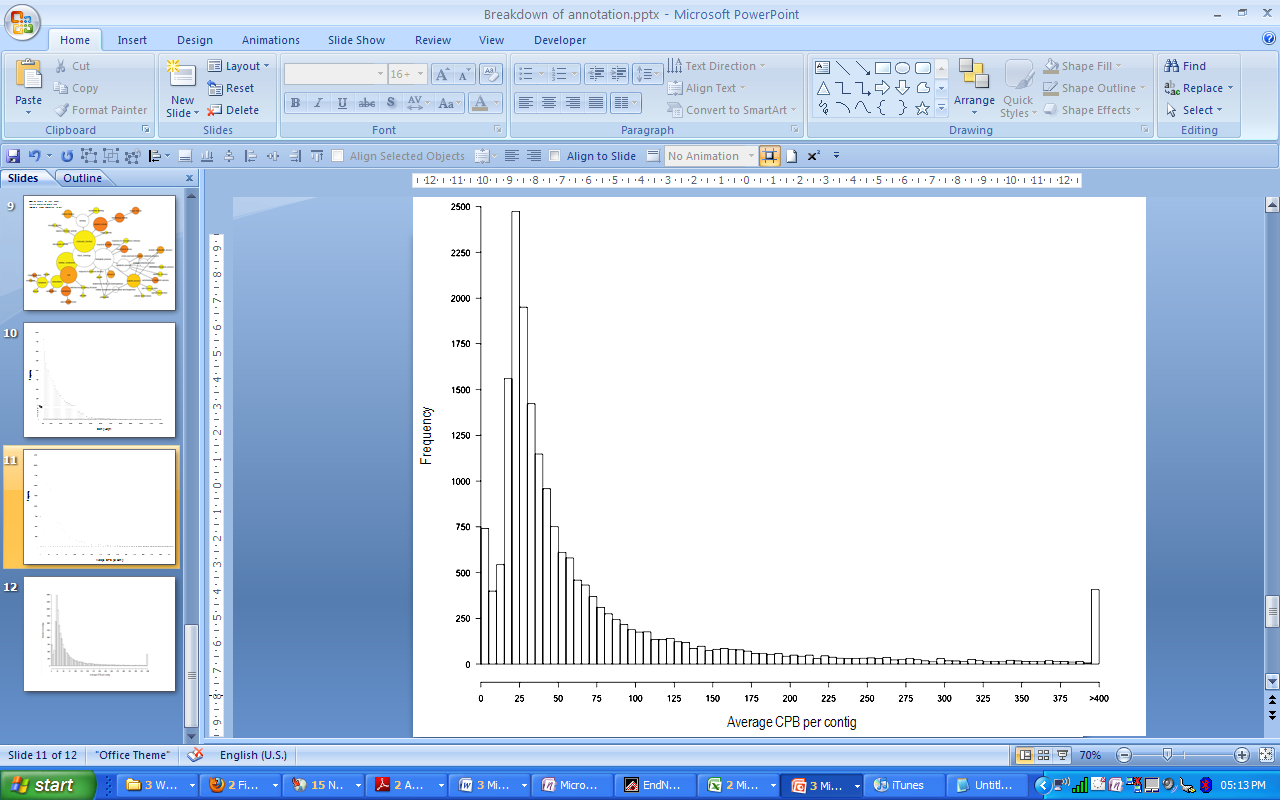
**

### Supplemental Figure S2.


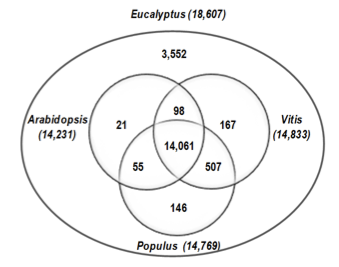


### Supplemental Figure S3.

### Supplemental Figure S4.

###
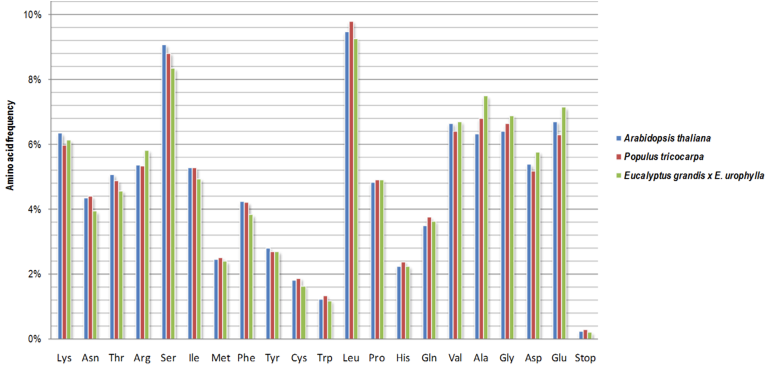
Supplemental Figure S5.

*Eucalyptus* hybrid

*Arabidopsis*

### Supplemental Figure S6.

### Supplemental Figure S7.

### Supplemental Figure S8.

# References

1. Ko JH, Beers EP, Han KH: **Global comparative transcriptome analysis identifies gene network regulating secondary xylem development in Arabidopsis thaliana**. *Mol Genet Genomics* 2006, **276**(6):517-531.

2. Ranik M, Myburg AA: **Six new cellulose synthase genes from *Eucalyptus* are associated with primary and secondary cell wall biosynthesis**. *Tree Physiol* 2006, **26**(5):545-556.

3. Zerbino DR, Birney E: **Velvet: Algorithms for de novo short read assembly using de Bruijn graphs**. *Genome Res* 2008, **18**(5):821-829.

4. Trapnell C, Williams BA, Pertea G, Mortazavi A, Kwan G, van Baren MJ, Salzberg SL, Wold BJ, Pachter L: **Transcript assembly and quantification by RNA-Seq reveals unannotated transcripts and isoform switching during cell differentiation**. *Nat Biotechnol* 2010, **28**(5):511-515.

5. Pinheiro M, Afreixo V, Moura G, Freitas A, Santos MAS, Oliveira JL: **Statistical, computational and visualization methodologies to unveil gene primary structure features**. *Met Inf Med* 2006, **45**(2):163-168.

6. Burge C, Karlin S: **Prediction of complete gene structures in human genomic DNA**. *J Mol Biol* 1997, **268**(1):78-94.
