# Supplementary material for: De novo assembled expressed gene catalog of a fast-growing Eucalyptus tree produced by Illumina mRNA-Seq
Source: BMC Genomics. 2010 Dec 1;11:681. doi: 10.1186/1471-2164-11-681 (PMC3053591; doi:10.1186/1471-2164-11-681)
Supplement: Additional file 3 — Contig validation, Needleman-Wunsch alignment figures. [file 1471-2164-11-681-S3.DOC]

## Additional file 3

**Contig validation**

The validity of the assembled contigs was evaluated by the alignment and coverage per base of the assembled contig and its respective CDS with the full length *Eucalyptus* reference present in NCBI. The coverage per base for the sequences was calculated using BWA (Li and Durbin 2009) and a global pairwise alignment between the sequences was performed using the needle package from EMBOSS (Rice et al. 2000). Plots from the generated alignments were constructed with the coverage associated with every base on the y-axis of the plot. Zero coverage values were assigned to the gaps in the alignments. This graph then indicated whether gaps and/or misassembled regions are present within the assembled contig, and to what depth level these contigs were sequenced.


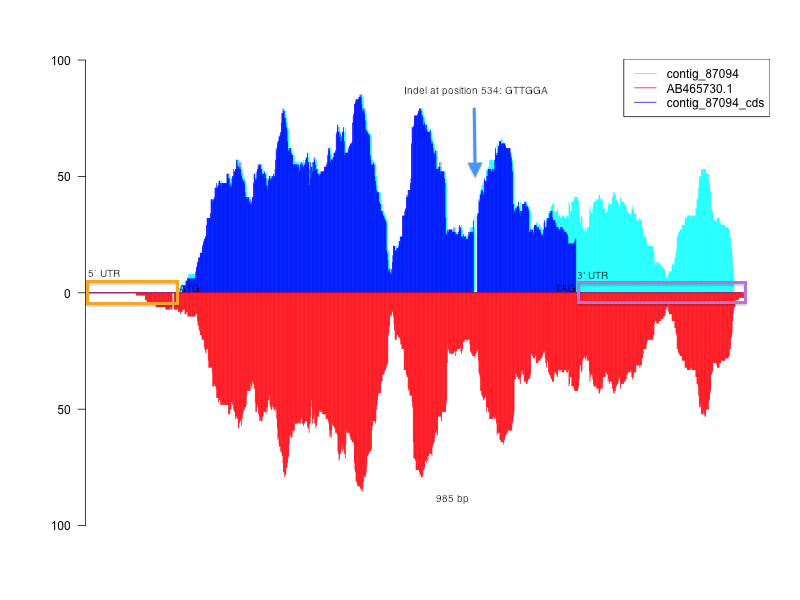


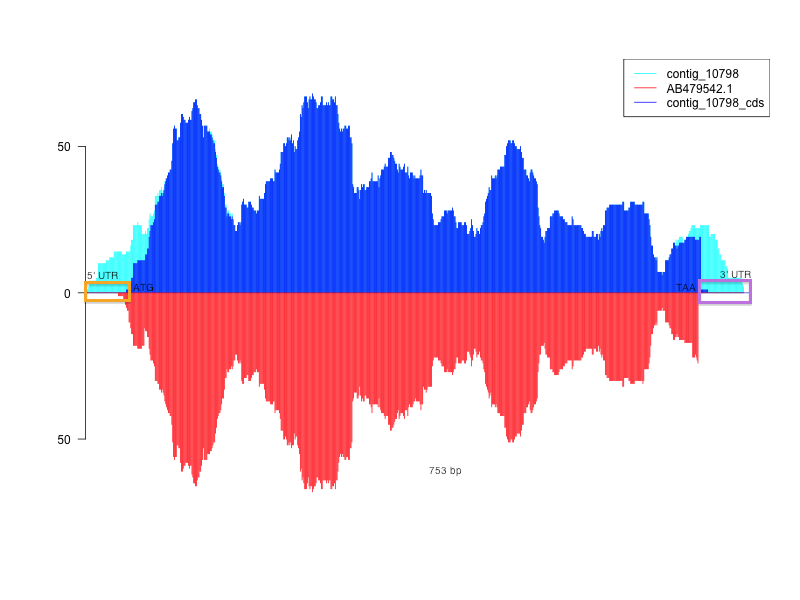


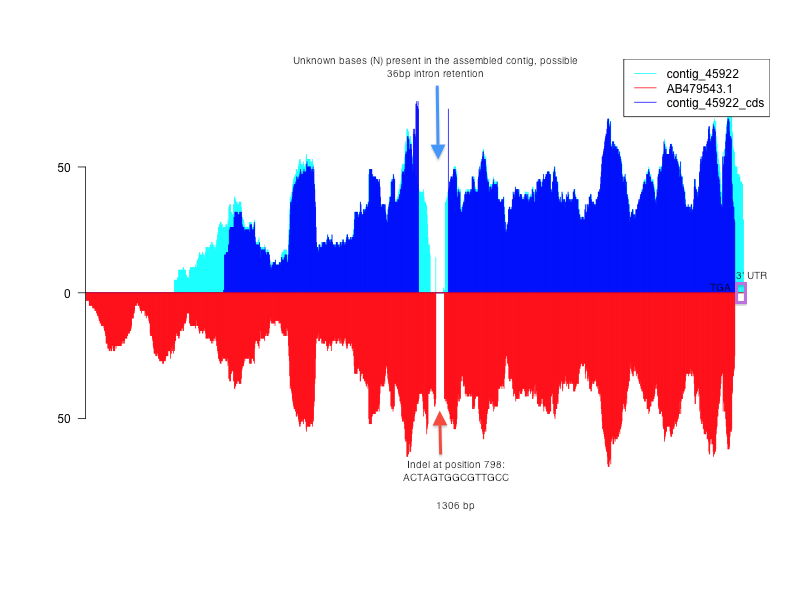


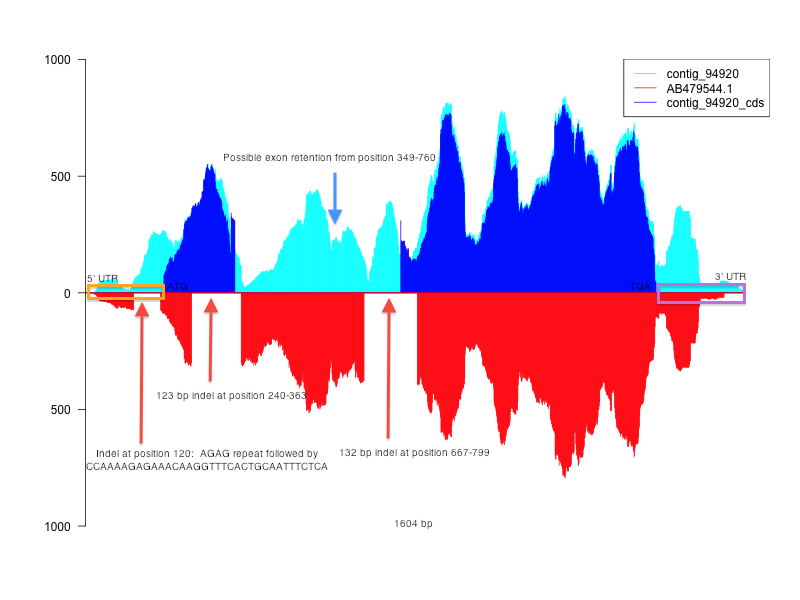


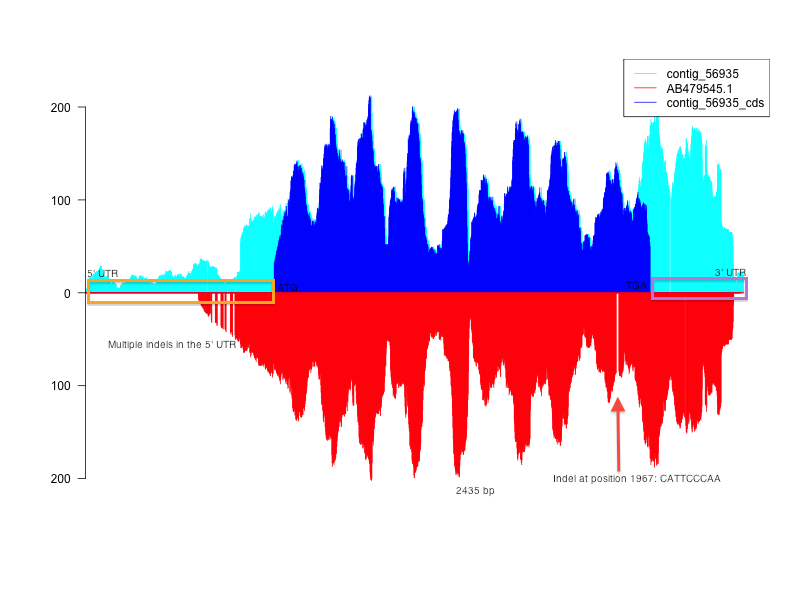


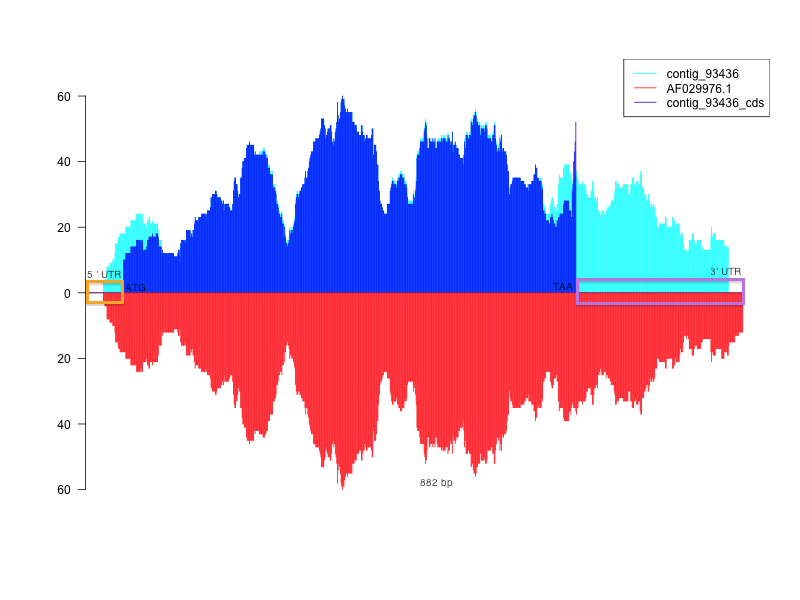


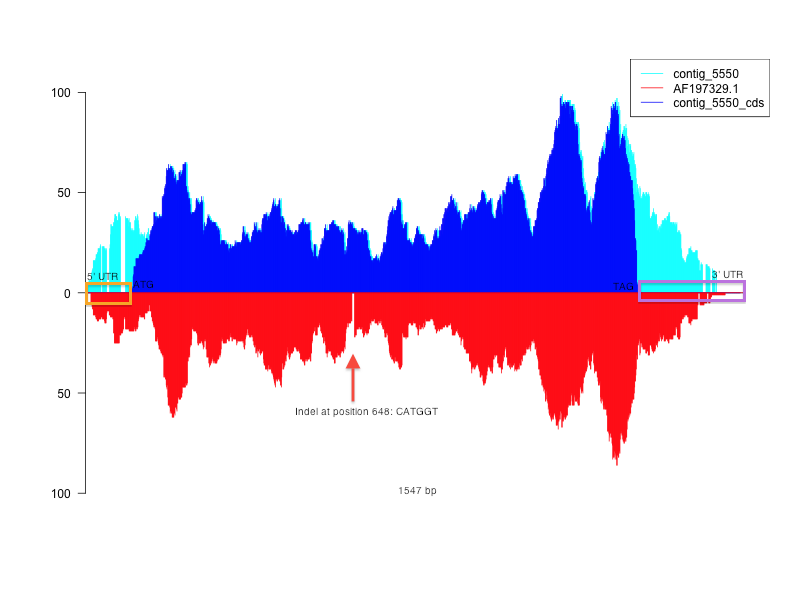


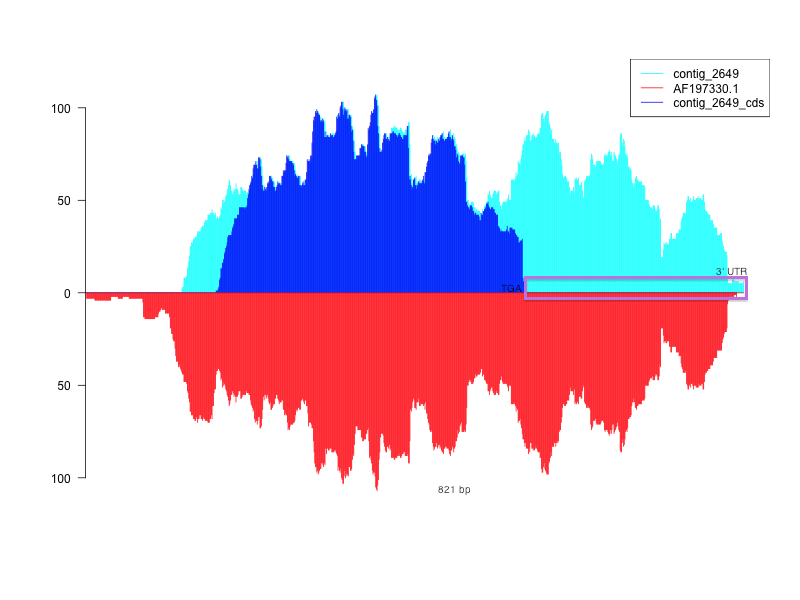


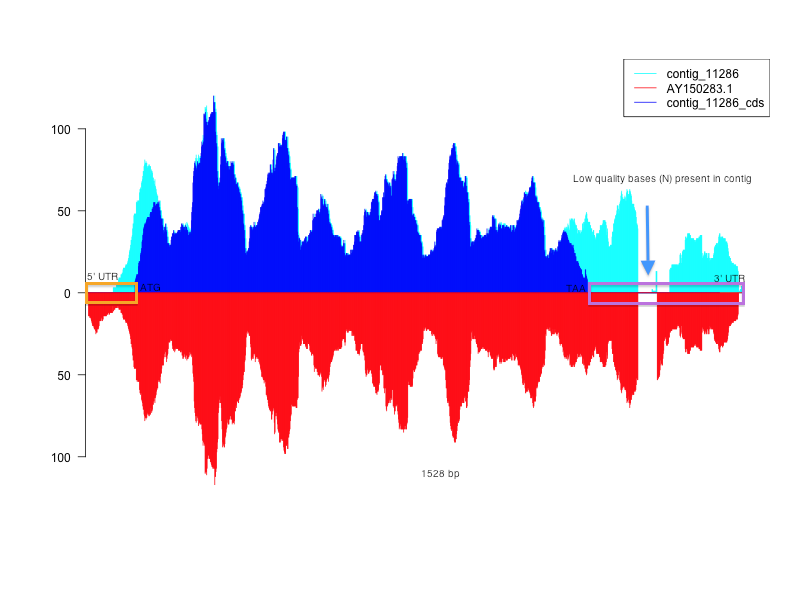


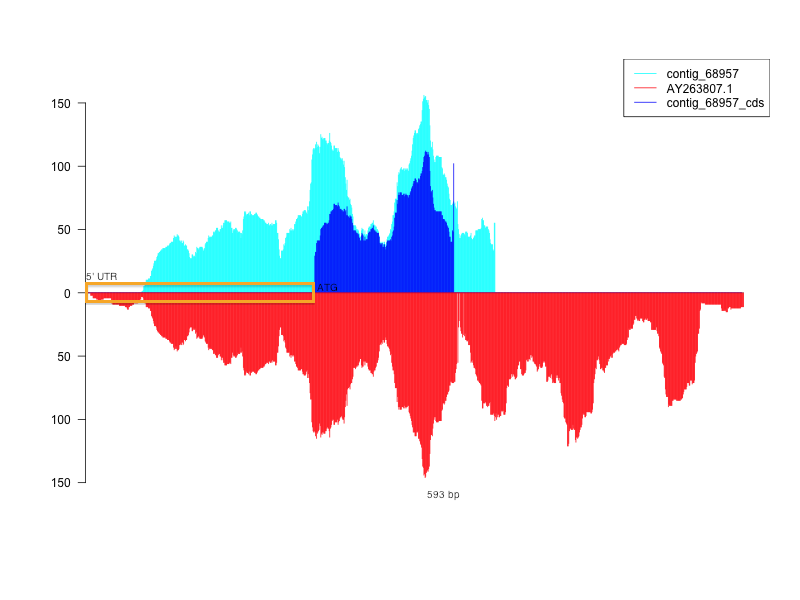


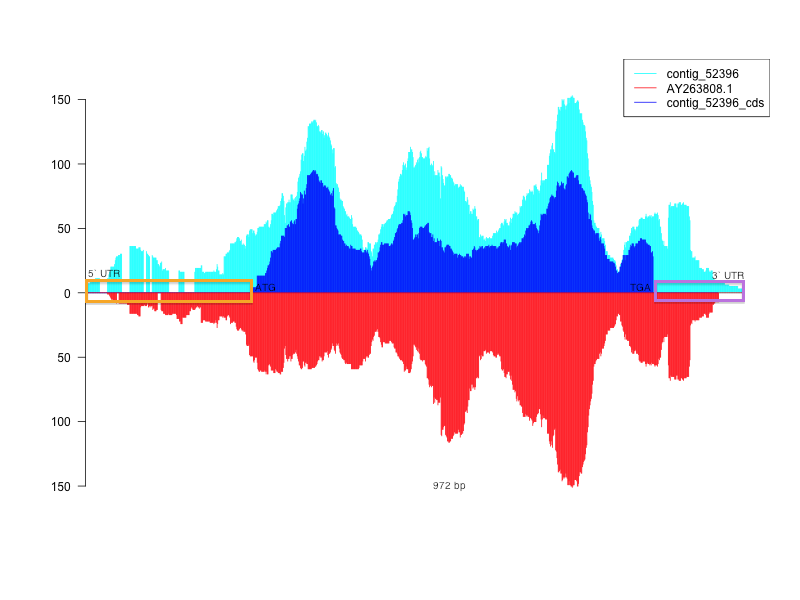

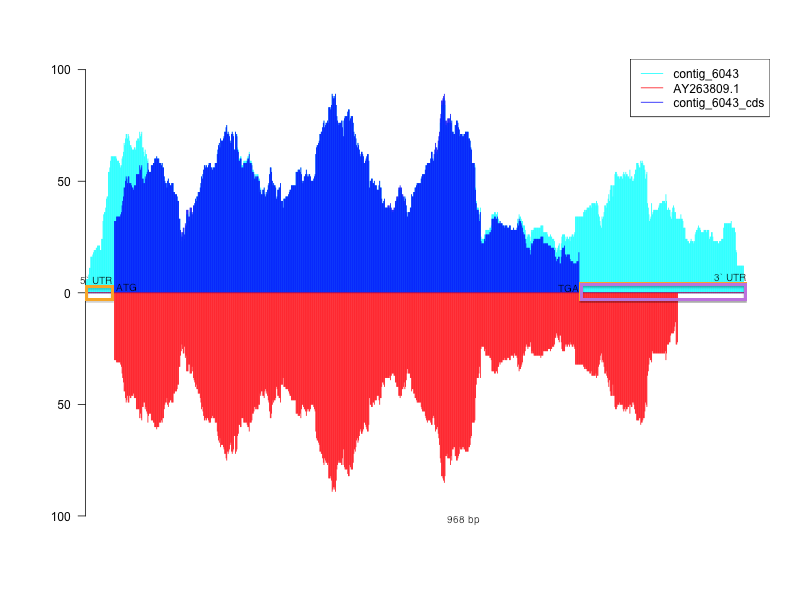


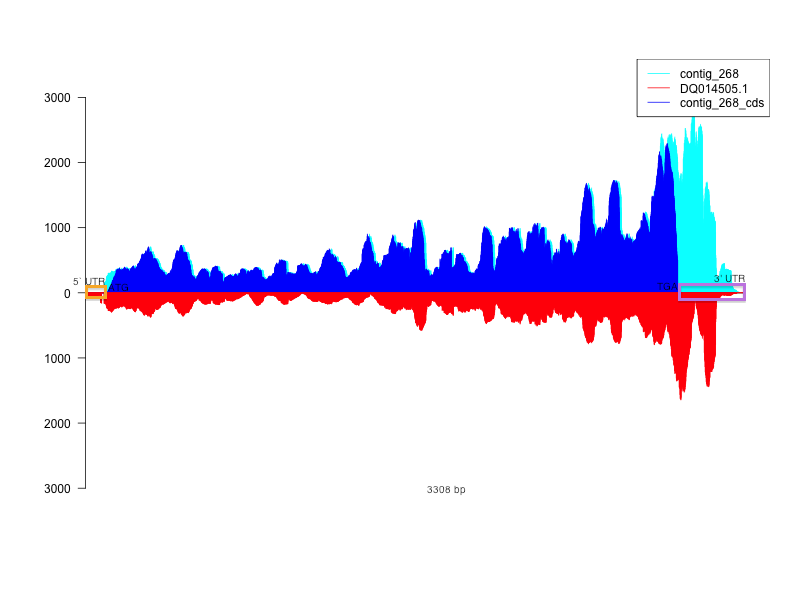

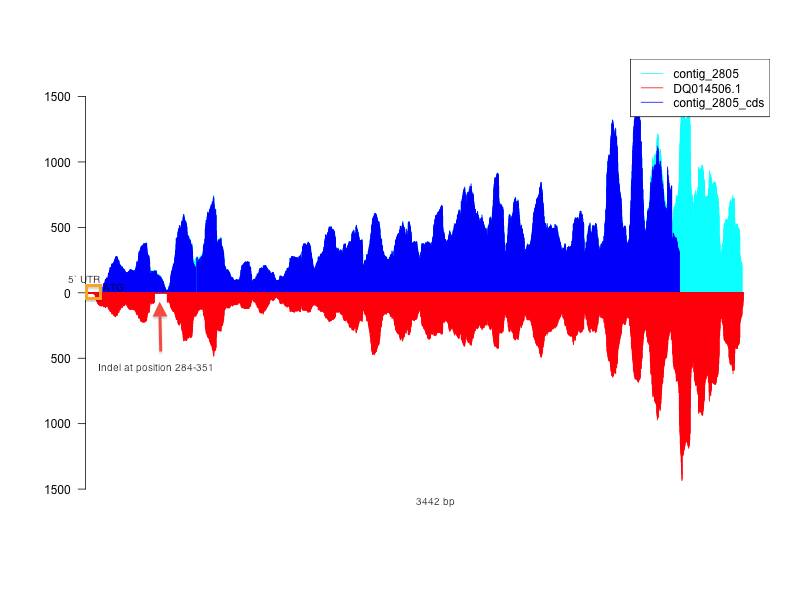


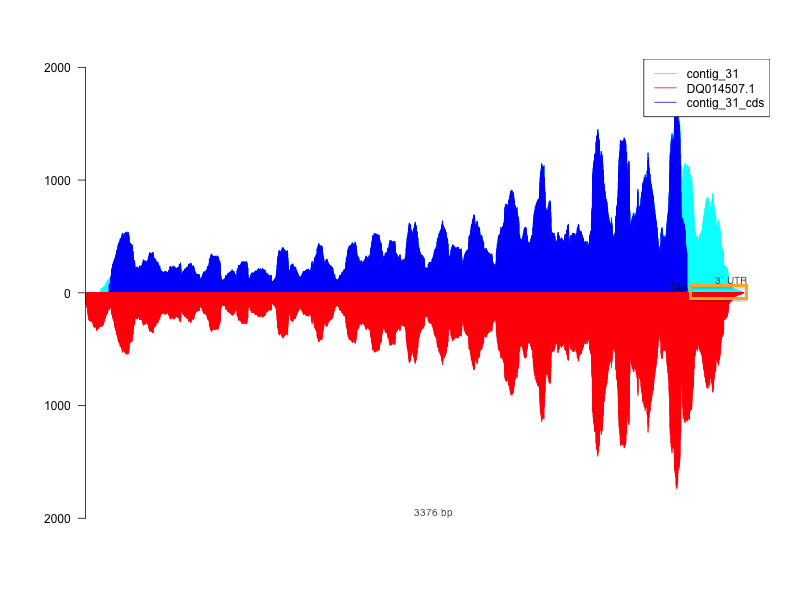


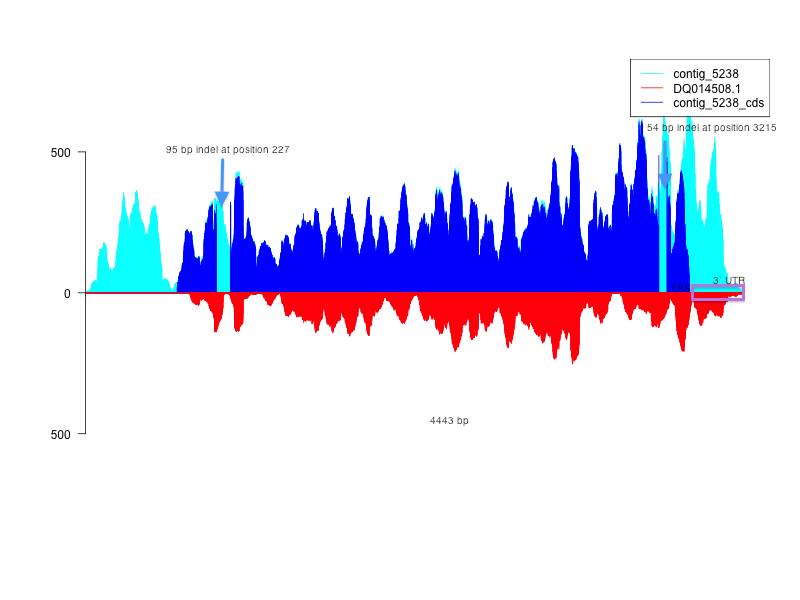


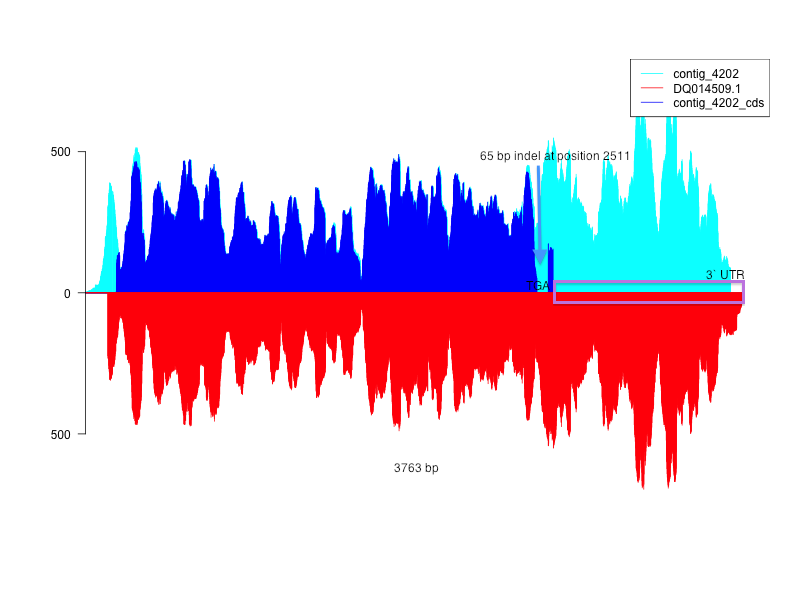


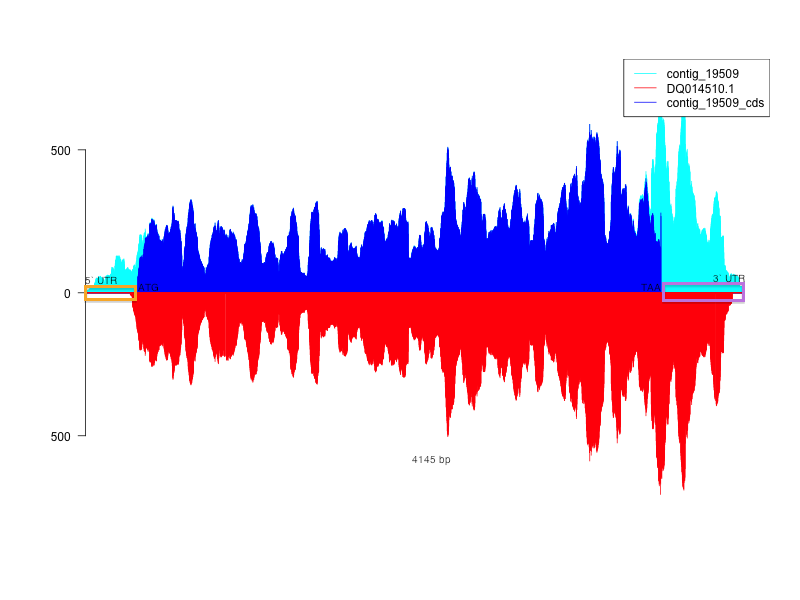


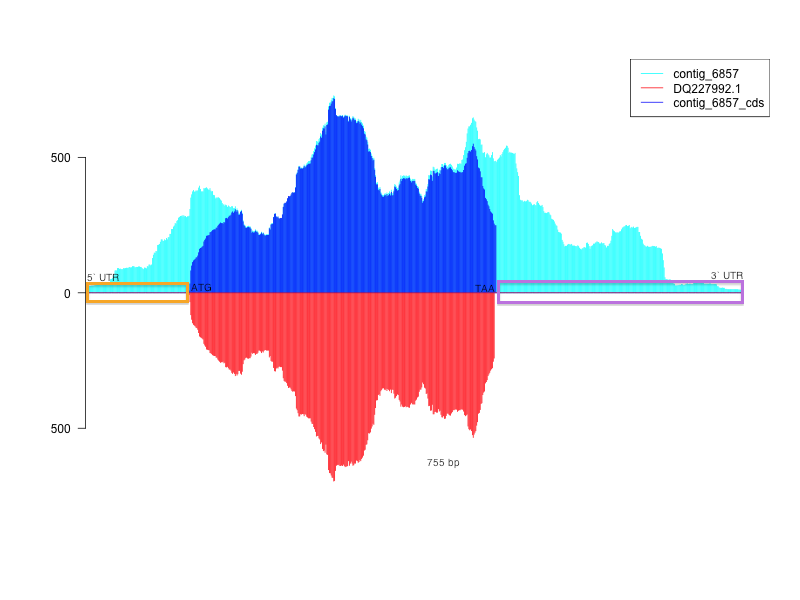

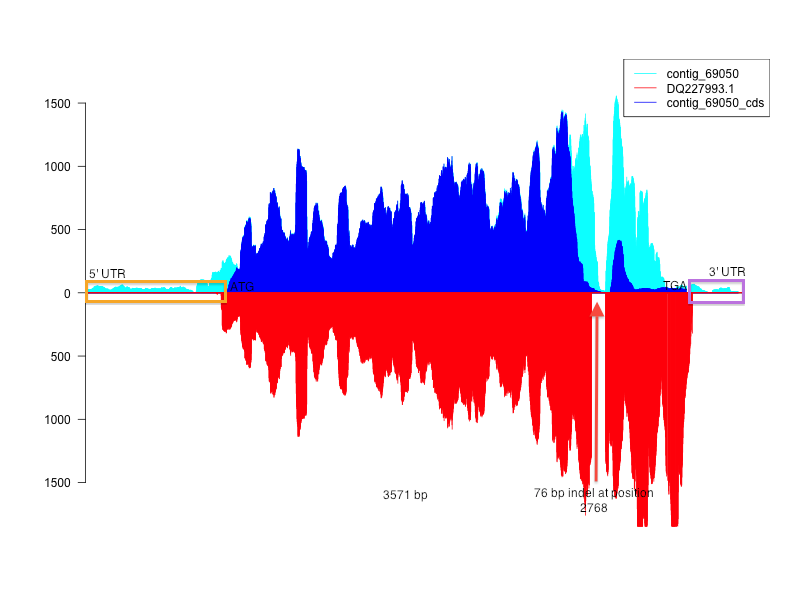

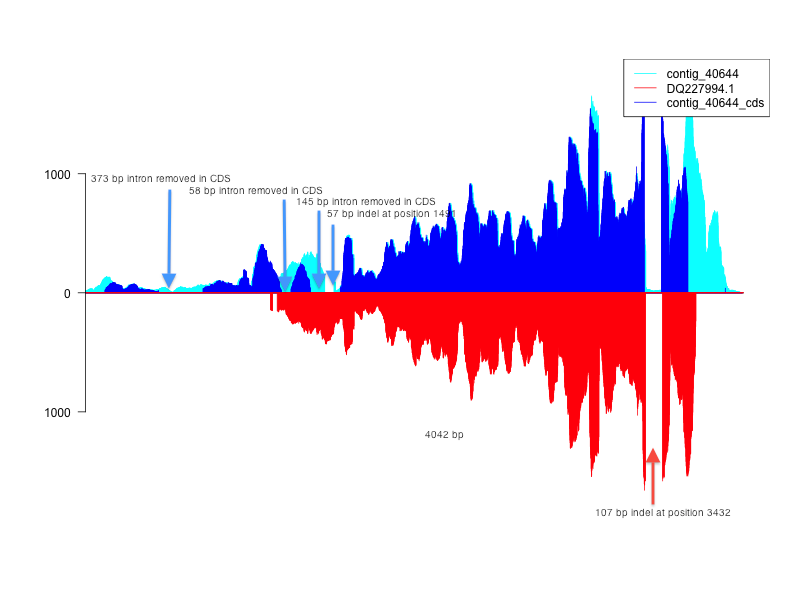


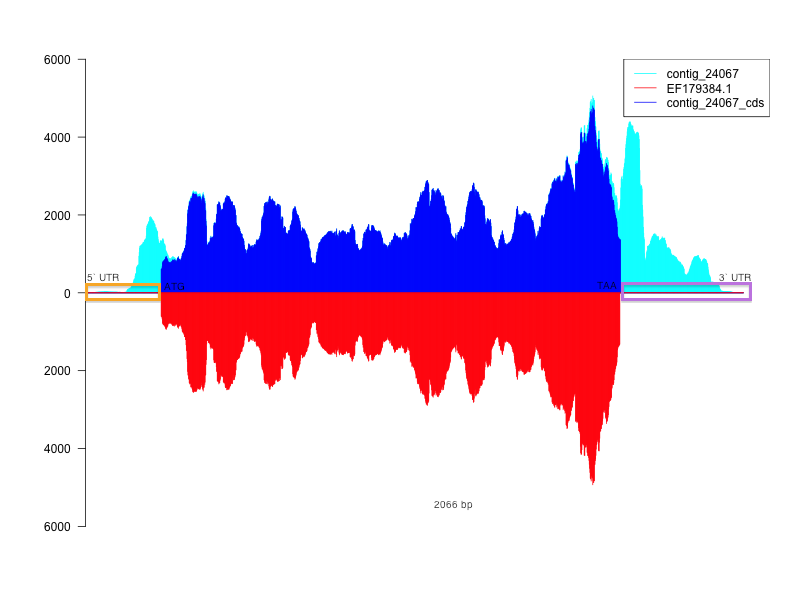


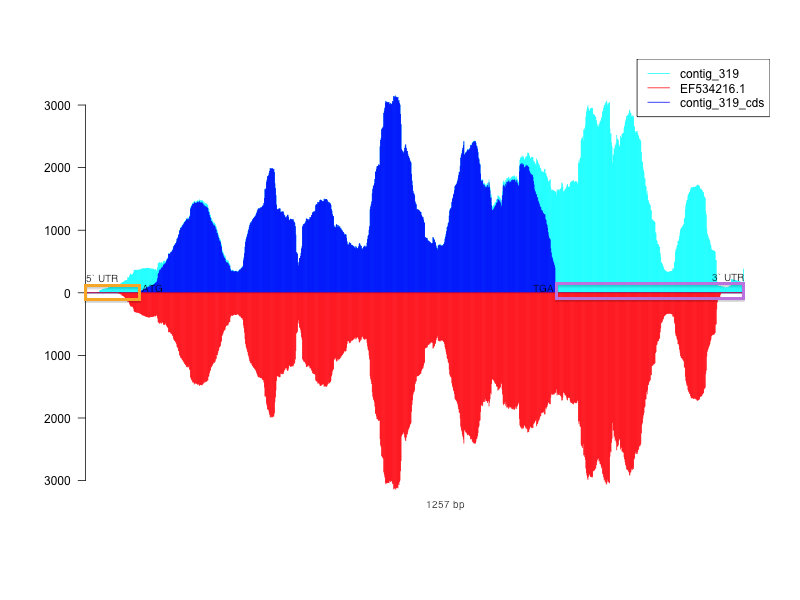


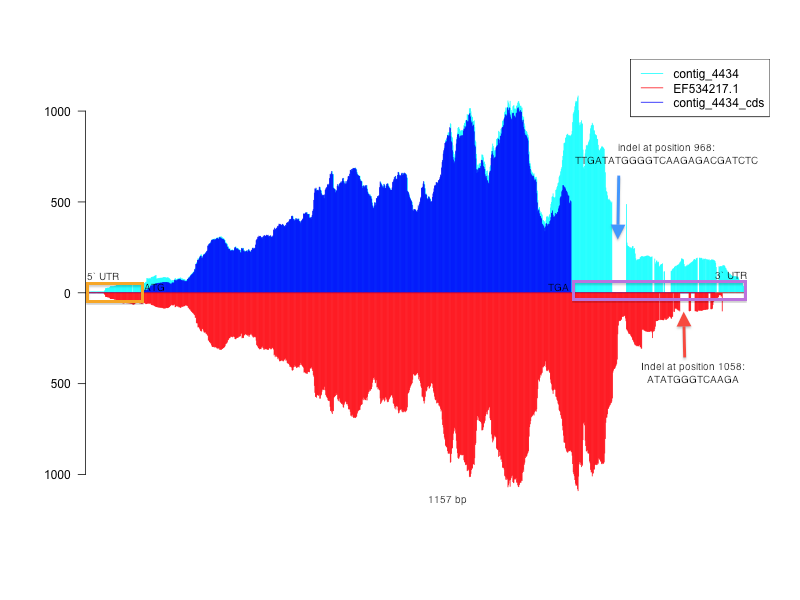


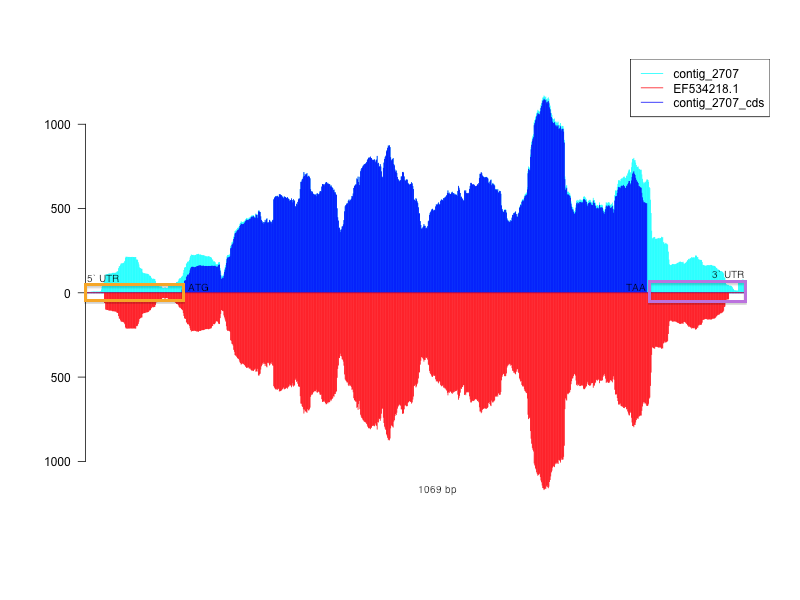


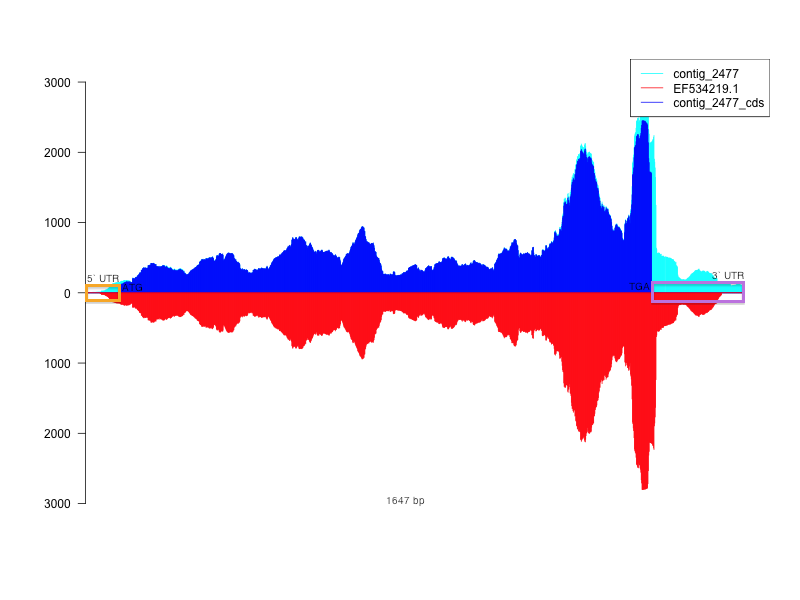


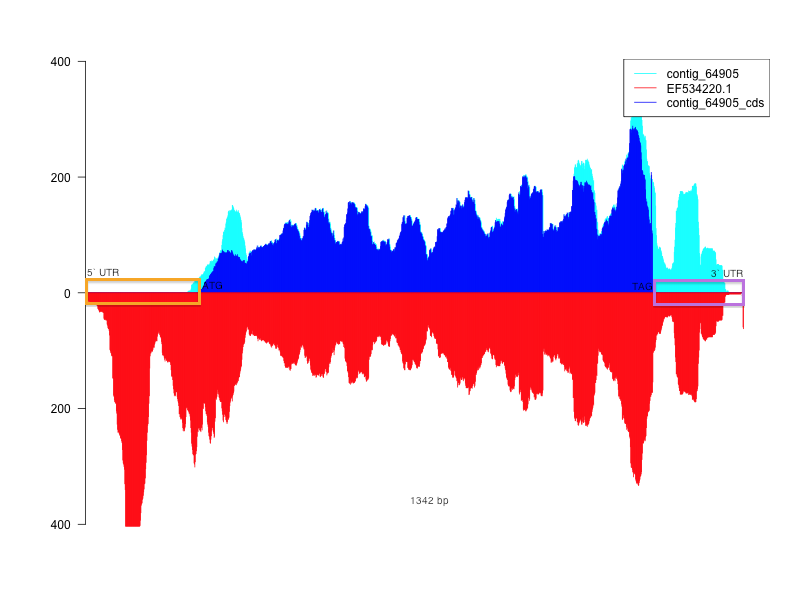


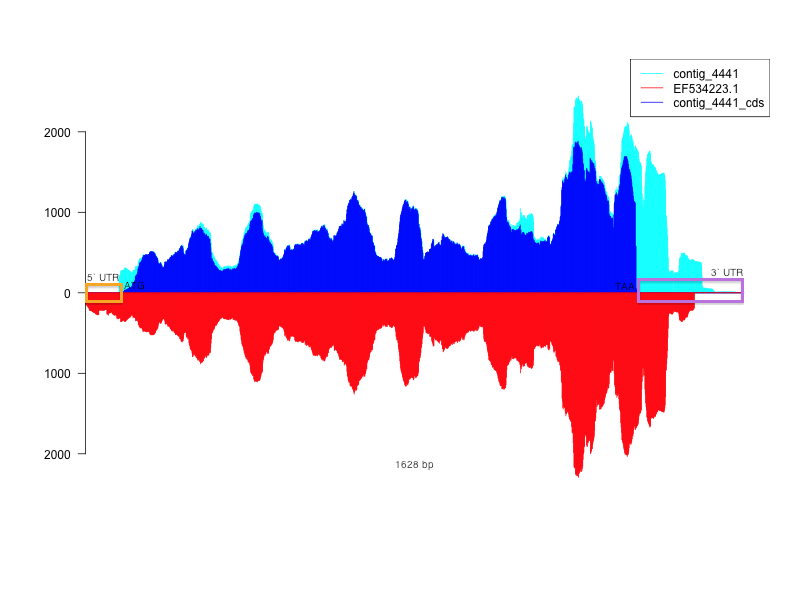


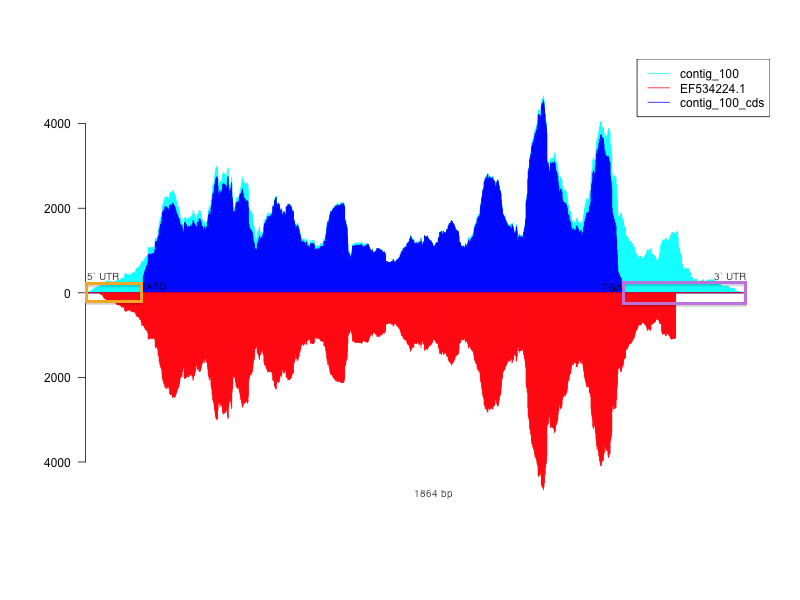


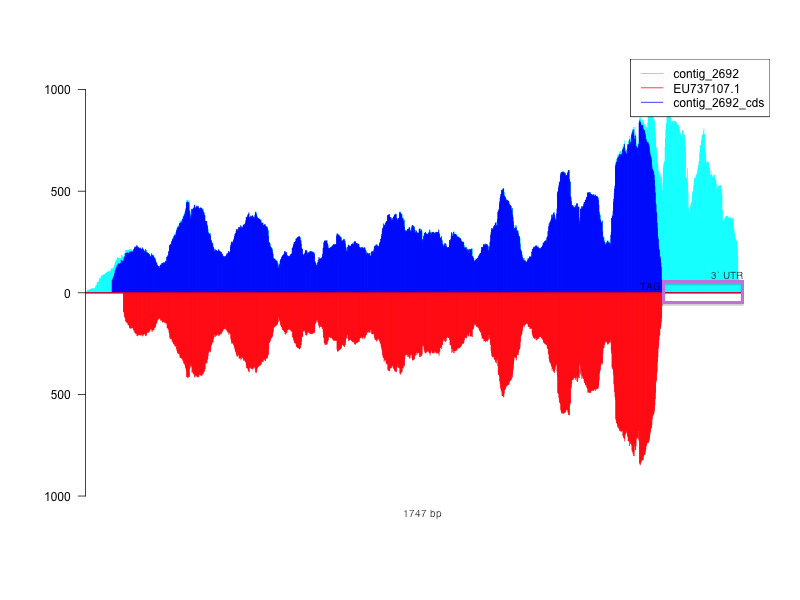


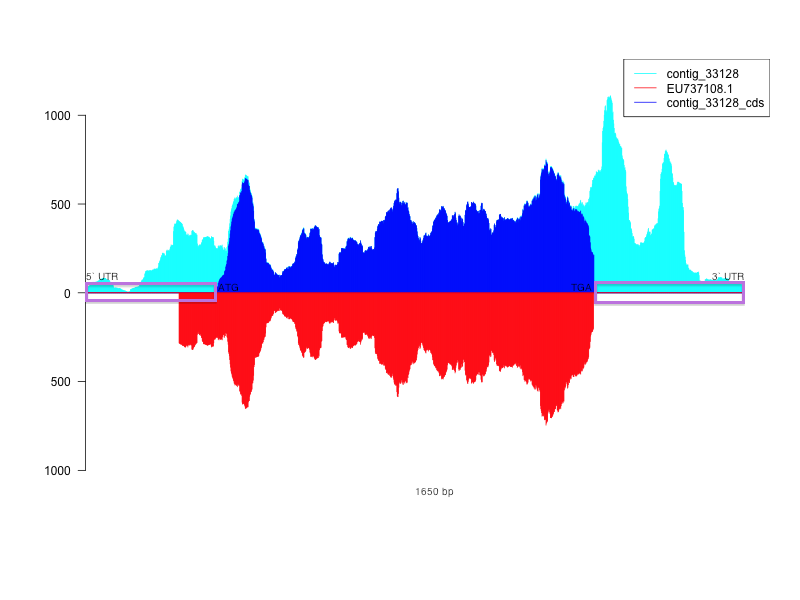


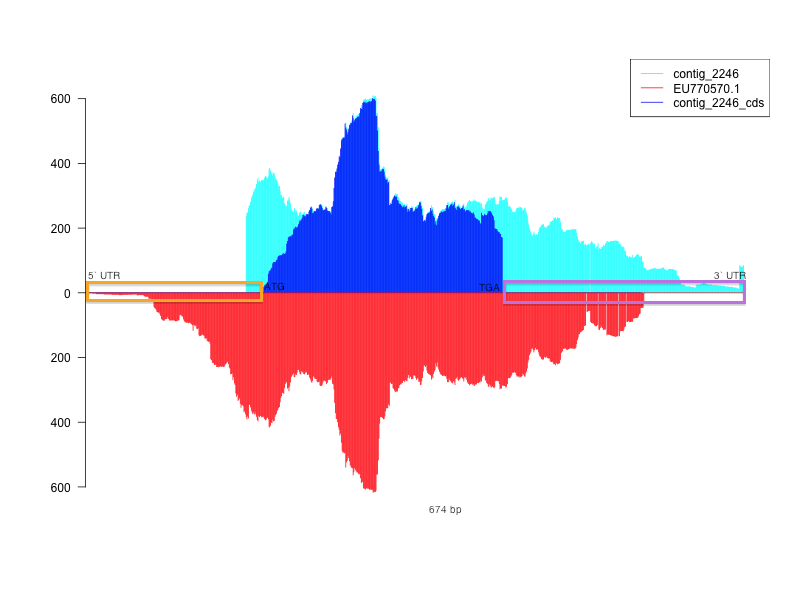


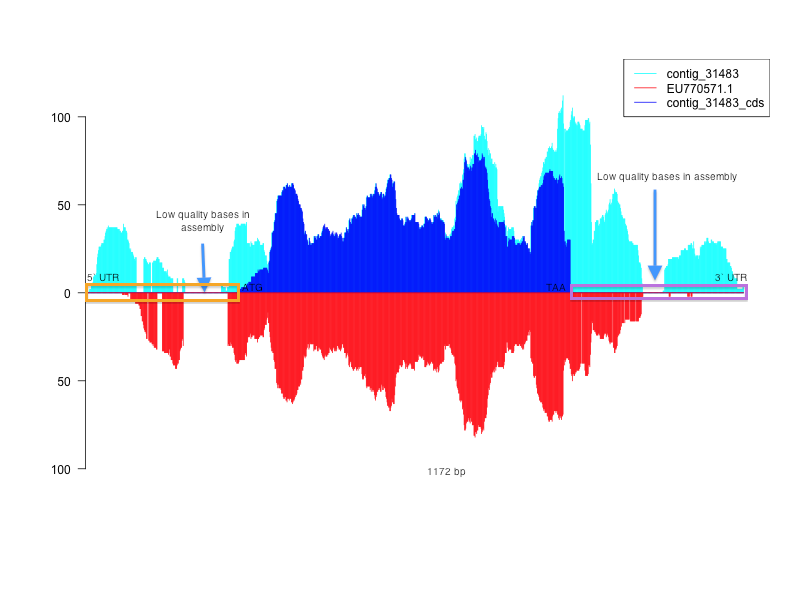


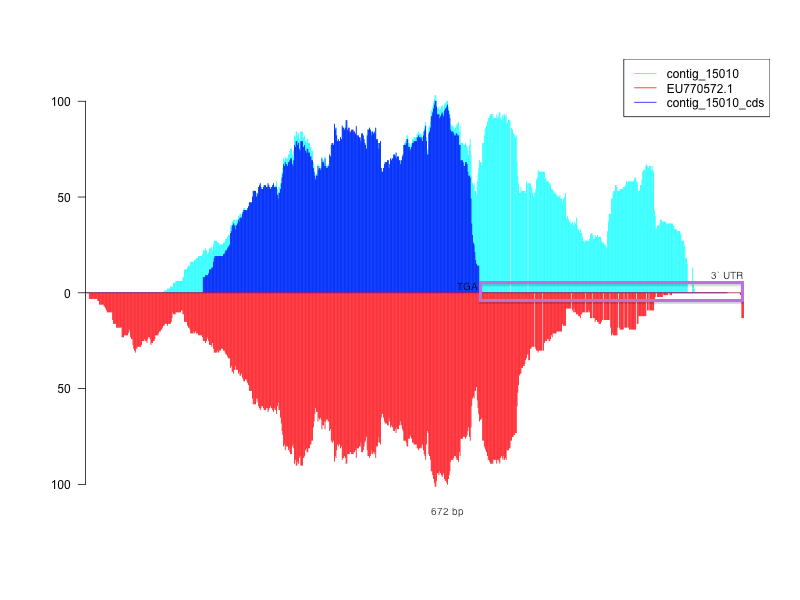


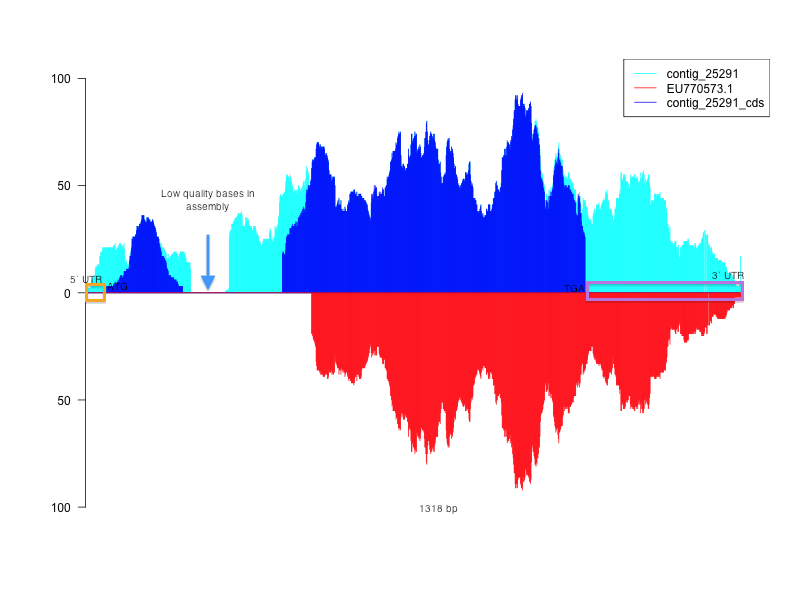


Li, H. and R. Durbin. 2009. Fast and accurate short read alignment with Burrows-Wheeler transform. *Bioinformatics* **25:** 1754.

Rice, P., I. Longden, and A. Bleasby. 2000. EMBOSS: the European molecular biology open software suite. *Trends in genetics* **16:** 276-277.
